# Measurement of precarious work in Brazil: translation, adaptation and dimensionality of the *Employment Precariousness Scale* (EPRES-Br)

**DOI:** 10.1590/0102-311XEN206125

**Published:** 2026-08-21

**Authors:** Victor Rocha Santana, Alejandra Vives-Vergara, Gláucio Roberto Santana de Jesus, Rita de Cássia Pereira Fernandes

**Affiliations:** 1 Instituto de Saúde Coletiva, Universidade Federal da Bahia, Salvador, Brasil.; 2 Universidad Católica de Chile, Santiago, Chile.; 3 Programa de Pós-graduação em Saúde, Ambiente e Trabalho, Universidade Federal da Bahia, Salvador, Brasil.

**Keywords:** Precarious Employment, Psychometrics, Surveillance of the Workers Health, Validation Study, Epidemiology and Biostatistics, Empleo Precario, Psicometría, Vigilancia de la Salud del Trabajador, Estudio de Validación, Epidemiología y Bioestadística

## Abstract

This study analyzed the psychometric properties - acceptability, reliability, and factorial structure - of the Brazilian version of the *Employment Precariousness Scale* (EPRES-Br), applied to two samples of Brazilian workers: workers with diseases that have been notified compulsorily by the Brazilian Unified National Health System in Salvador and physical therapists in Bahia. Overall, 638 individuals with formal and informal work contracts answered the instrument. The acceptability, internal consistency, and dimensionality of the scale were evaluated using descriptive and analytical statistics, including factor analysis with methodology for categorical variables, appropriate to ordinal data and based on polychoric correlations. The EPRES-Br showed high acceptability (above 90%) and acceptable internal consistency (Cronbach’s alpha = 0.77 for workers in Salvador and 0.84 for physical therapists). The dimensions Wages, Disempowerment and Vulnerability confirmed the theoretical construct, while Temporariness and Rights carried the same factor, and two items on time off carried outside the original theoretical dimension (Exercise Rights). It is concluded that EPRES-Br has good psychometric properties for use in epidemiological studies in Brazil, although revisions of some items are necessary, considering their meaning for the context of the world of work.

## Resumen

Este estudio analizó las propiedades psicométricas -aceptabilidad, fiabilidad y estructura factorial- de la versión brasileña de la *Escala de Precariedad Laboral* (EPRES-Br), que se aplicó a dos muestras de trabajadores brasileños: trabajadores con agravios de notificación obligatoria por el Sistema Único de Salud en Salvador y fisioterapeutas en Bahía. En total, 638 personas con vínculos laborales formales e informales respondieron al instrumento. La aceptabilidad, la consistencia interna y la dimensionalidad de la escala se evaluaron mediante estadística descriptiva y analítica, incluyendo el análisis factorial con metodología para variables categóricas, adecuada para datos ordinales y basada en correlaciones policóricas. La EPRES-Br presentó una alta aceptabilidad (superior al 90%) y una consistencia interna aceptable (alfa de Cronbach = 0,77 para los trabajadores de Salvador y 0,84 para los fisioterapeutas). Las dimensiones Remuneración, Desempoderamiento y Vulnerabilidad confirmaron el constructo teórico, mientras que Temporalidad y Derechos cargaron en el mismo factor, y dos ítems acerca de días libres cargaron fuera de la dimensión teórica original (Ejercicio de los Derechos). Se concluye que la EPRES-Br presenta buenas propiedades psicométricas para su uso en estudios epidemiológicos en Brasil, aunque es necesario revisar algunos ítems, teniendo en cuenta su significado en el contexto del mundo del trabajador.

## Introduction

Precarious work is a social determinant of health, linked by evidence to mental health problems, musculoskeletal disorders and occupational accidents ^1^ ^,^ ^2^. Furthermore, precarious work affects both informal and formal employment relationships, leading to uncertainty, insecurity, ambiguous employment arrangements, restricted rights, low wages and limited opportunities for collective representation of labor rights ^3^. These conditions contribute to situations of social vulnerability.

Despite the interest in investigating precarious work, theoretical models addressing its multidimensional nature have emerged only recently ^2^ ^,^ ^4^. Likewise, the development of a measurement instrument capable of capturing this multidimensionality began just over a decade ago, with initial work conducted in Spain, followed by validation studies of the instrument ^5^ ^,^ ^6^ ^,^ ^7^ ^,^ ^8^ ^,^ ^9^.

The history of precarious work displacing standard employment in Western industrialized countries is well documented ^1^. However, in Brazil, the universalization of wage employment with guaranteed labor rights, full-time working hours and collective bargaining was never fully consolidated, given the systematic exclusion of women and Black workers through a structural surplus of labor, conditions analogous to slavery and unpaid work (particularly in the care economy) ^10^ ^,^ ^11^. Consequently, more recent forms of precarious work ^12^ have been added to pre-existing forms that remain unresolved.

Classified by the World Bank as an upper-middle-income country, Brazil ranks among the world’s eight largest economies ^13^. In April 2024, the seasonally adjusted unemployment rate stood at 7.5% and was on a downward trend, with 100.8 million employed persons in a country of 203 million inhabitants. Among those employed in 2023, 39.4 million workers (39.2%) were engaged in informal employment ^14^. Nevertheless, despite the highest level of formal employment contracts recorded over the past 12 years, half of these formal jobs end within 24 months ^15^.

The health and economic crisis caused by the COVID-19 pandemic further exacerbated these internal contradictions. As a result, increased worker vulnerability has led to rising poverty and precarious work, in a self-perpetuating cycle of reinforcement ^1^ ^,^ ^16^.

Although no universally accepted definition of precarious work has yet been established, prevailing definitions generally describe it as employment characterized by insecurity regarding job continuity, insufficient income and the absence of social and labor protection ^4^.

Studies investigating the association between precarious work and adverse mental health outcomes have reported positive associations, particularly when multidimensional measures of exposure were used. Studies relying on unidimensional measures, such as perceived job insecurity or temporary employment, showed limited sensitivity and have therefore been discouraged in future research ^17^.

The *Employment Precariousness Scale* (EPRES), developed by Amable ^5^, is used to measure the multidimensional nature of precarious wage employment in epidemiological studies. It comprises six dimensions and 22 items ^18^. Seven studies assessing its psychometric properties have been identified, conducted in five countries (Spain, Sweden, Chile, Greece and Germany) and in a region including six Central American countries ^19^ ^,^ ^20^ ^,^ ^21^ ^,^ ^22^ ^,^ ^23^ ^,^ ^24^. Following an initial validation study based on a 26-item version ^19^, Spain conducted a new validation study in 2015 ^25^ using the second version of the EPRES, introduced in 2010 (EPRES II), which contains 22 items.

Overall, these validation studies supported the theoretical framework of the EPRES, confirming its six dimensions. The only exception was the Central American study, which administered and analyzed items from only four dimensions, although its findings remained consistent with those of the other studies.

To date, no studies conducted in Brazil have used the EPRES or a translation and adaptation of this instrument to Portuguese and the Brazilian context, despite the country’s high levels of labor precariousness and substantial burden of work-related illness ^2^. As a consequence, estimating the population exposed to precarious work remains challenging, limiting the development of new public policies. Accordingly, an instrument for measuring precarious work should contribute to greater conceptual and methodological convergence, thereby enabling comparisons across countries.

Given the above, we used the EPRES in two studies conducted in 2023. The instrument was translated into Brazilian Portuguese (EPRES-Br) and cross-culturally adapted to the context of the Brazilian labor market. Given the high degree of heterogeneity and informality of this market, and considering discussions by the scale’s developers, the instrument was administered to workers with different employment arrangements, evaluating the extent to which its theoretical framework still holds in this context ^26^. The main goal of this study was to assess the acceptability, reliability and dimensionality of the EPRES-Br. Secondary objectives included characterizing the study samples, describing the translation and cross-cultural adaptation process, and evaluating the performance of the factors corresponding to the dimensions of the original theoretical construct. Also, based on the accumulated evidence from previous validation studies, we hypothesized that the items comprising the Temporariness dimension might exhibit weaker psychometric properties in a context characterized by higher levels of informal labor.

## Methods

### Translation and adaptation of the EPRES-Br

(1) To achieve the objectives of this study, the EPRES II-2010 ^18^ was translated from English (version provided by the Health and Ecology Inequalities Research Group - Employment Conditions Network [GREDS-EMCONET] at the Pompeu Fabra University, Spain, https://www.upf.edu/web/greds-emconet/) into Brazilian Portuguese, followed by cross-cultural adaptation and adjustment to the Brazilian labor market, as described as follows.

(2) For the item concerning income, originally defined as monthly net earnings, participants were instructed to consider not only income from work but also ongoing social benefits, such as the Brazilian Income Transfer Program. This adaptation was introduced to better reflect the objective socioeconomic conditions of workers in more vulnerable settings, where multiple sources of income may contribute to the resources available for social reproduction.

(3) The translation was carried out within the Epidemiology, Health Surveillance and Labor Studies research group (Brazilian National Research Council - CNPq, acronym in Portuguese) by three bilingual researchers: an epidemiologist and two graduate students from the Federal University of Bahia (UFBA, acronym in Portuguese). These coauthors of this article are health professionals based in the state of Bahia, and the research group has extensive experience in occupational epidemiology both in local and nationwide contexts.

(4) The back-translation of the Portuguese version into English was performed by a native English-speaking language instructor from England, fluent in Portuguese.

(5) Subsequently, three independent researchers - an epidemiologist and two social scientists specializing in workers’ health - reviewed the translated versions, examining both the Brazilian Portuguese translation and the back-translated English text.

(6) Upon completion of this stage, and to further improve the appropriateness of the language, the instrument was administered to formal and informal workers affiliated with the Domestic Workers’ Union of Bahia. These workers were selected because the researchers had access to the union. At the end of the process, participants were invited to provide open-ended feedback on their experience completing the instrument. Although the number of participants was small, the results were considered valuable, as the respondents demonstrated a satisfactory understanding of the meaning of the questions and the response options.

### Participants and study design

The study involving workers with health conditions subject to mandatory reporting within the Brazilian Unified National Health System (SUS, acronym in Portuguese) aimed to test the hypothesis that precarious work is associated with self-directed violence (a concept encompassing suicidal ideation, suicidal behavior and self-harm) ^27^. This was a case-control study, the results of which have been submitted for publication. Participants were selected from the Brazilian Information System for Notifiable Diseases (SINAN, acronym in Portuguese) based on reports of self-directed violence (case group) and human post-exposure rabies prophylaxis (control group). Inclusion criteria were deriving income from their own work, having an accessible telephone number and being at least 18 years of age. Exclusion criteria included death and being a student or retired. All eligible cases with an available telephone number were contacted, whereas potential controls were selected through simple random sampling at a ratio of two controls for every case. Participants were interviewed by telephone by trained interviewers, previously certified for the study. A total of 180 individuals were interviewed from a pool of 402 eligible participants, yielding a response rate of 44.8%.

The study involving physical therapists in the state of Bahia investigated factors associated with precarious work in this professional group. This was a cross-sectional study conducted through a web-based survey, the results of which have also been submitted for publication. The inclusion criteria were being a practicing physical therapist in the state of Bahia, with income derived from such practice. A total of 458 fully completed questionnaires were obtained.

As is common in online surveys, the questionnaire link was disseminated through multiple channels by both the research team and physical therapists serving as service coordinators, who were asked to share the link with workgroups and individual contacts via WhatsApp and to encourage colleagues to forward it to other physical therapists. Consequently, it was not possible to determine how many physical therapists received the survey link or to calculate a response rate, which is an inherent limitation of online surveys. Although it cannot be guaranteed that all eligible professionals were informed about the study, the combined dissemination strategies achieved broad outreach within the physical therapy community, as internet access is universal among these professionals. In this context, there is no indication that non-participation was attributable to any specific factor, i.e., there is no evidence suggesting meaningful differences between respondents and non-respondents. Nevertheless, the use of convenience sampling should be acknowledged as a potential limitation, since the representativeness of the sample cannot be assured.

Accordingly, the total study population comprised 638 workers across the two samples. Data collection for both studies took place in 2023 as part of a master’s thesis in Health, Environment and Work and a doctoral dissertation in Public Health. Some degree of selection bias cannot be ruled out, given the challenges in accessing the case group - whose members had experienced the severe health outcome of self-directed violence in the study conducted among the population of Salvador - as well as the online nature of the survey administered to physical therapists.

### Statistical analysis

The EPRES-Br assessed the 22 items of the revised EPRES II-2010 across its six dimensions: Temporariness (two items), Disempowerment (two items), Vulnerability (five items), Wages (three items), Rights (four items) and Exercise Rights (six items). The response scale also followed the original instrument, with five ordered response categories for all dimensions except Rights, which comprised three ordered response categories ^18^.

As specified by the developers of the original scale, the subscore for each of the six dimensions was calculated as the mean of the responses to the corresponding items, resulting in a score ranging from 0 to 4. The overall score was calculated as the mean of the six dimension scores and likewise ranged from 0 to 4, with 0 representing the lowest possible level of employment precariousness ^18^.

In the initial statistical analysis, the EPRES-Br items were described using means and standard deviations, with missing data reported. Subsequently, Spearman item-dimension correlations were calculated for the items within each dimension, excluding the item under analysis from the corresponding dimension score to avoid overlap. Floor and ceiling effects - i.e., the proportions of respondents obtaining the lowest and highest possible scores - were also examined. Internal consistency reliability was assessed using Cronbach’s alpha coefficient ^28^. Missing data were not imputed. Whenever applicable, the number of valid observations (n) was reported for each item or dimension, with any variation reflecting missing data.

Categorical-variable-methodology factor analysis (CVM-FA) was conducted separately for each of the two study samples to explore the factor structure of the EPRES-Br ^29^. The analysis was based on a polychoric correlation matrix, using the robust diagonally weighted least squares (RDWLS) extraction method ^28^. The number of factors to retain was based on the original theoretical model and determined through parallel analysis using 500 bootstrap random samples ^18^ ^,^ ^30^. Robust promin rotation was applied ^31^. Parallel analysis had also been employed in one of the previous EPRES validation studies ^20^. For this stage of the analysis, respondents with at least one missing value were excluded, and a complete-case approach was adopted.

Model fit was evaluated using the root mean square error of approximation (RMSEA), the comparative fit index (CFI) and the Tucker-Lewis index (TLI). RMSEA values below 0.08 and CFI and TLI above 0.90 were considered acceptable fit criteria ^32^.

Model stability was assessed using the H-index ^29^. This index evaluates the extent to which a set of items represents a common factor and ranges from 0 to 1. Values greater than 0.80 indicate a well-defined latent variable that is likely to be replicated in future studies, whereas lower values suggest a poorly defined latent variable with lower expected replicability. Pratt’s importance measure ^33^ was also used to interpret potential discrepancies between cross-loadings when factor loadings were equal to or greater than of |0.30|. This measure indicates the proportion of variance in each item explained by each factor. Reliability was assessed using Cronbach’s alpha, with values of 0.70 or higher considered acceptable.

Descriptive analyses were performed using Stata, version 14.2 (https://www.stata.com), whereas exploratory factor analysis was conducted using FACTOR, version 12.04.05 (https://psico.fcep.urv.cat/utilitats/factor/index.html) ^34^. FACTOR is freely available software specifically designed for exploratory factor analysis (EFA/CVM-FA) and semi-confirmatory factor analysis (SCFA).

All participants provided written informed consent before participating in the study. The data were anonymized and both studies were approved by the Research Ethics Committee of the Bahia Medical School, Federal University of Bahia (approval numbers 5.258.142 and 6.046.587).

## Results

### Translation and adaptation of the EPRES-Br

The translation and adaptation of the EPRES II-2010 to the Brazilian context required the following adjustments: in Question 1 (“the duration of your contract is”), the response category “permanent contract” was supplemented with explanatory terms appropriate to the Brazilian context - formally registered employment (CLT) and civil service employment through competitive examination, in parentheses. In the Wages dimension (Questions 10-12), the term “remuneration” was added as a broader synonym, resulting in the wording “Your wages (or remuneration)”. This adaptation was intended to include respondents who earned income from work but did not receive formal wages. In Question 12, which concerns net income, respondents were instructed that this amount should include wages (or remuneration) and social benefits (Brazilian Income Transfer Program) ^35^. In addition, the values originally expressed in euros in the English version of the scale were converted into Brazilian minimum wages (in *reais*) based on the 2022 minimum wage value (Supplementary Material; https://cadernos.ensp.fiocruz.br/static//arquivo/suppl-e00206125_3895.pdf).

### Study participants

Predominant among the participants were women, individuals of non-White race/skin color, and workers exposed to moderate to high employment precariousness (EPRES-Br > 1.30 among workers in Salvador and > 1.29 among physical therapists). Workers identified through the SUS had lower levels of education, whereas all physical therapists held university degrees. More than half of both the Salvador workers and the physical therapists had permanent employment contracts (54.8% and 59.9%, respectively). Nevertheless, there was substantial participation of workers in informal or temporary employment arrangements. Overall, approximately 70% of the participants had been employed by their current employer for less than 10 years, with shorter job tenure observed among those with temporary contracts (Table 1).

**Table 1:** Sociodemographic and occupational characteristics of workers in Salvador and physical therapists in Bahia. Brazil, 2023..

Characteristics	Workers in Salvador (N = 180)		Physical therapists in Bahia (N = 458)	
	n	%	n	%
Sex				
Women	114	63.3	330	72.0
Men	66	36.7	128	28.0
Race/Skin color				
Yellow/White	26	14.4	140	31.0
Indigenous/Mixed-race/Black	154	85.6	316	69.0
Educational level *				
Higher	30	16.7	405	88.4
Lower	150	83.3	53	11.6
Marital status and children				
Single with children/Married/Living with partner	118	65.6	269	58.7
Single	62	34.4	189	41.3
Employment relationship **				
Standard employment	120	67.0	356	77.9
Precarious employment	59	33.0	101	22.1
Length of employment (years)				
≥ 5	89	49.7	236	54.5
< 5	90	50.3	197	45.5

Note: the number of valid observations may vary across variables due to missing data.

* Educational level (dichotomized): workers in Salvador - higher: complete secondary or tertiary education; lower: from illiteracy to incomplete secondary education; physical therapists - higher: specialization, master’s degree or doctorate; lower: undergraduate degree;

** Employment relationship (dichotomized): standard employment (workers in Salvador): formally registered employment or voluntary self-employment; precarious employment (workers in Salvador): unregistered employment or involuntary self-employment; standard employment (physical therapists): public sector employment. formally registered employment, business ownership or voluntary legal entity status; precarious employment (physical therapists): temporary contract or involuntary legal entity status.

The occupations represented in the sample of workers identified through the SUS included healthcare (15.2%), retail (6.3%), cleaning services (5%), transportation (5%) and domestic work (5.6%), among other less frequently represented occupations.

Participants identified through the SUS ranged in age from 18 to 77 years, with a median age of 39.5 years. Among the physical therapists, ages ranged from 23 to 70 years, with a median age of 37 years.

### Psychometric properties of the EPRES-Br

Table 2 presents the proportion of missing data, means, standard deviations, response frequencies and item-dimension correlations for each item. Among the workers in Salvador, the response rate was high (greater than 90%) and all response options were used. In general, each item showed a stronger correlation with its corresponding dimension than with the other dimensions, with the exception of Q1, Q5 and Q13 (Table 2).

**Table 2:** Descriptive statistics of items from the Brazilian version of the *Employment Precariousness Scale* (EPRES-Br) and Spearman item-dimension correlations. Workers with health conditions reported to the Brazilian Unified National Health System (SUS) in Salvador, Bahia State, Brazil, 2023.

Dimension/Item	Missing data (%)	Mean	SD	Frequency of response categories (%)					Spearman item-dimension correlation					
				0	1	2	3	4	Temporariness	Disempowerment	Vulnerability	Wages	Rights	Exercise Rights
Temporariness														
Contract duration	7.78	0.90	1.11	50.6	8.3	27.7	2.8	2.8	0.26	0.06	-0.09	0.15	0.48	0.24
Length of employment	0.56	1.64	1.40	28.9	20.6	21.1	15.0	13.8	0.26	0.15	0.04	0.16	0.19	0.11
Disempowerment														
Defines schedule	1.67	2.04	1.54	20.0	6.7	28.3	36.1	7.2	0.15	0.59	-0.03	0.01	0.07	0.14
Defines wages	1.67	2.18	1.33	21.7	2.8	21.7	41.1	11.0	0.12	0.59	-0.03	-0.03	0.16	0.18
Vulnerability														
Exposure to disciplinary action	1.11	1.96	1.64	33.3	6.7	17.8	12.8	28.3	0.00	-0.03	0.08	0.19	-0.07	0.11
Feeling vulnerable	0.00	1.00	1.21	48.3	20.6	20.0	4.4	6.7	0.00	-0.02	0.50	0.33	0.07	0.16
Risk of dismissal	1.67	0.94	1.35	55.0	19.4	10.0	2.2	11.7	0.02	-0.02	0.47	0.18	0.05	0.17
Authoritarian treatment	1.11	0.83	1.18	59.4	11.7	18.4	4.4	5.0	0.00	-0.02	0.54	0.34	0.15	0.23
Feeling replaceable	1.67	1.66	1.52	32.8	16.1	23.9	3.3	22.2	-0.09	0.02	0.34	0.05	-0.08	0.08
Wages														
Basic expenses	0.00	1.63	1.43	34.4	10.0	26.7	15.6	13.3	0.14	0.02	0.19	0.70	0.21	0.25
Unforeseen expenses	0.56	2.53	1.31	11.0	8.9	25.6	23.9	30.0	0.09	0.01	0.31	0.65	0.21	0.22
Net income	1.11	3.19	1.03	1.1	6.7	17.8	20.6	52.7	0.36	0.02	0.25	0.55	0.28	0.11
Rights														
Retirement	1.11	0.58	0.71	54.4	31.7	12.8	NA	NA	0.40	0.16	0.04	0.26	0.40	0.11
Severance	1.67	0.85	0.73	35.0	43.3	20.0	NA	NA	0.13	0.11	-0.02	0.13	0.42	0.14
Maternity leave	1.67	0.51	0.62	55.0	36.7	6.6	NA	NA	0.40	0.09	0.05	0.26	0.48	0.28
Insurance	1.11	0.66	0.58	38.9	54.4	5.6	NA	NA	0.18	-0.02	-0.06	0.06	0.44	0.20
Exercise Rights														
Weekly day off	1.11	0.86	1.28	63.3	6.1	15.0	8.9	5.6	0.11	0.09	0.13	0.17	0.13	0.47
Medical certificate	1.11	1.43	1.57	49.4	2.8	15.6	17.2	13.9	0.10	0.11	0.19	0.20	0.23	0.56
Medical appointment	1.11	1.01	1.25	55.0	7.2	18.9	16.1	1.7	0.20	0.17	0.15	0.18	0.14	0.72
Paid vacation	1.11	1.36	1.64	54.4	2.8	11.7	11.7	18.3	0.30	0.18	0.03	0.08	0.38	0.60
Time off for personal reasons	1.67	1.69	1.48	36.1	5.6	22.2	21.7	12.7	0.09	0.03	0.17	0.19	0.04	0.40
Time off for family reasons	1.67	1.76	1.53	36.7	3.9	20.0	22.2	15.5	0.18	0.17	0.14	0.20	0.12	0.59

Note: correlations highlighted in bold indicate each set of items corresponding to the respective theoretical construct; n per item may vary due to missing data.

NA: not applicable; SD: standard deviation.

Among the physical therapists (Table 3), the response rate was likewise high (> 90%) and respondents used all response options for each item. Overall, item-dimension correlations were stronger with the corresponding dimension than with the others, except for Q1 (contract duration), Q12 (net income), Q15 (maternity leave) and Q19 (paid vacation) (Table 3).

**Table 3:** Descriptive statistics of items from the Brazilian version of the *Employment Precariousness Scale* (EPRES-Br) and Spearman item-dimension correlations. Physical therapists in Bahia, Brazil, 2023.

Dimension/Item	Missing data (%)	Mean	SD	Frequency of response categories (%)					Spearman item-dimension correlation					
				0	1	2	3	4	Temporariness	Disempowerment	Vulnerability	Wages	Rights	Exercise Rights
Temporariness														
Contract duration	8.08	0.62	0.94	59.9	11.2	18.2	1.3	1.3	0.42	1.14	-0.07	0.09	0.57	0.22
Length of employment	5.46	1.50	1.22	23.5	27.9	22.5	13.6	7.0	0.42	0.13	0.06	0.30	0.37	0.20
Disempowerment														
Defines schedule	5.90	1.88	1.24	19.8	13.8	22.7	33.2	4.6	0.11	0.55	0.08	0.12	0.21	0.13
Defines wages	6.11	2.03	1.39	26.8	3.2	8.7	50.4	4.8	0.19	0.55	0.09	0.14	0.24	0.16
Vulnerability														
Exposure to disciplinary action	5.02	1.99	1.28	16.8	14.6	30.0	21.0	12.6	0.03	0.16	0.45	0.23	0.02	0.34
Feeling vulnerable	5.46	1.36	1.09	24.2	28.8	28.0	9.8	3.7	-0.06	0.07	0.68	0.33	-0.01	0.36
Risk of dismissal	5.24	1.41	1.14	21.6	34.6	23.1	9.0	6.5	0.00	0.07	0.50	0.24	-0.04	0.26
Authoritarian treatment	4.80	1.07	1.10	38.3	25.1	21.0	7.9	2.9	-0.06	0.01	0.63	0.32	-0.02	0.32
Feeling replaceable	4.80	1.92	1.36	17.5	21.7	25.6	12.3	18.1	0.04	0.11	0.65	0.32	0.03	0.33
Wages														
Basic expenses	1.97	1.88	1.18	13.3	23.8	32.7	17.7	10.5	0.09	0.12	0.36	0.68	0.09	0.35
Unforeseen expenses	2.40	2.72	1.09	3.5	9.0	27.7	28.8	28.6	0.05	0.04	0.36	0.67	0.08	0.27
Net income	1.53	2.47	0.97	3.3	11.0	34.0	37.0	13.2	0.51	0.18	0.19	0.36	0.37	0.23
Rights														
Retirement	2.18	0.57	0.79	60.4	19.2	18.2	NA	NA	0.56	0.26	0.03	0.25	0.59	0.25
Severance	2.84	0.93	0.85	38.4	27.3	31.5	NA	NA	0.25	0.16	0.11	0.21	0.44	0.23
Maternity leave	2.40	0.34	0.62	71.4	18.5	7.6	NA	NA	0.48	0.08	-0.05	0.06	0.44	0.20
Unemployment insurance	2.18	0.49	0.64	57.2	33.0	7.6	NA	NA	0.38	0.13	-0.12	0.03	0.53	0.17
Exercise Rights														
Weekly day off	3.06	1.14	1.20	41.8	17.0	24.9	8.6	4.6	0.18	0.10	0.15	0.16	0.27	0.49
Medical certificate	3.06	1.46	1.36	36.0	14.0	21.4	17.5	8.0	0.21	0.12	0.27	0.22	0.22	0.56
Medical appointment	2.40	1.51	1.24	29.8	17.4	26.6	19.0	4.8	0.16	0.18	0.41	0.29	0.15	0.66
Paid vacation	2.84	1.33	1.39	39.8	17.7	18.5	10.5	10.7	0.47	0.16	0.19	0.17	0.43	0.40
Time off for personal reasons	3.49	2.24	1.27	13.3	12.0	26.9	27.3	17.0	0.03	0.13	0.43	0.35	0.09	0.62
Time off for family reasons	3.71	2.12	1.25	14.5	13.5	28.2	26.4	13.7	0.03	0.08	0.42	0.32	0.07	0.62

Note: correlations highlighted in bold indicate each set of items corresponding to the respective theoretical construct; n per item may vary due to missing data.

NA: not applicable; SD: standard deviation.

Table 4 presents n, means, standard deviations, proportion of missing data, score ranges, floor and ceiling effects (%) and internal consistency (Cronbach’s alpha).

**Table 4:** Descriptive statistics of items from the Brazilian version of the *Employment Precariousness Scale* (EPRES-Br): mean, standard deviation (SD), range (floor and ceiling effects) and internal consistency (Cronbach’s alpha). Workers with health conditions reported to the Brazilian Unified National Health System (SUS) in Salvador, and physical therapists in Bahia, Brazil, 2023.

Dimensions	EPRES items	n	Mean	SD	Missing data (%)	Interval	Floor (%)	Ceiling (%)	Cronbach’s alpha
Workers reported to SUS									
Temporariness	1 and 2	166	1.29	1.01	7.8	0-4	17.2	1.7	0.43
Disempowerment	3 and 4	176	2.11	1.15	2.2	0-4	13.9	6.1	0.75
Vulnerability	5 to 9	174	1.29	0.89	3.3	0-4	5.6	0.6	0.73
Wages	10 to 12	178	2.46	1.06	1.1	0-4	1.1	7.8	0.79
Rights	13 to 16	176	1.31	0.91	2.2	0-4	20.0	1.1	0.63
Exercise Rights	17 to 22	177	1.34	0.99	0.1	0-4	17.2	0.6	0.77
EPRES-Br	1 to 22	158	1.62	0.55	12.2	0.52-3.04	0.6	0.6	0.77
Physical therapists									
Temporariness	1 and 2	421	1.07	0.92	8.08	0-4	19.0	1.3	0.60
Disempowerment	3 and 4	429	1.96	1.17	6.33	0-4	16.4	3.1	0.73
Vulnerability	5 to 9	429	1.55	0.89	6.33	0-4	3.7	0.9	0.80
Wages	10 to 12	447	2.36	0.88	2.40	0-4	0.7	2.8	0.74
Rights	13 to 16	442	1.17	1.06	3.49	0-4	30.8	2.6	0.70
Exercise Rights	17 to 22	432	1.63	0.92	5.68	0-4	5.7	0.7	0.81
EPRES-Br	1 to 22	399	1.62	0.58	12.88	0.22-3.19	0.2	0.2	0.84

Note: alpha values ≥ 0.70 were considered acceptable; floor and ceiling effects correspond to the proportion of respondents at the minimum and maximum scale values, respectively.

Among the workers in Salvador, subscale scores ranged from 1.29 for Temporariness to 2.46 for Wages. The overall EPRES-Br score was 1.62. Floor effects were below 5.7% for the Vulnerability and Wages dimensions but exceeded 13% for the remaining dimensions. Ceiling effects were generally low (< 5%), except for Disempowerment (6.1%) and Wages (7.8%). Cronbach’s alpha coefficients were generally above 0.74, with the exception of Rights (0.60) and Temporariness (0.50). The overall Cronbach’s alpha for the scale was 0.77 (Table 4) ^36^.

Among the physical therapists, subscale scores ranged from 1.07 for Temporariness to 2.36 for Wages. The overall EPRES-Br score was likewise 1.62. Floor effects were below 5.7% for the Vulnerability, Exercise Rights and Wages dimensions, but exceeded 16.38% for Temporariness, Disempowerment and Rights. Ceiling effects were low (< 5%). Cronbach’s alpha coefficients were 0.70 or higher for all dimensions except Temporariness (0.60). The overall Cronbach’s alpha for the scale was 0.84 (Table 4) ^36^.

### Factor analysis

The factor composition and corresponding factor loadings are presented in Table 5, which also reports the proportion of variance explained by factor, Cronbach’s alpha coefficients, and H, RMSEA, CFI and TLI indices.

Among the workers in Salvador (n = 158), all items exhibited factor loadings greater than 0.30 across the six factors. The item “medical appointment” showed cross-loadings on the Exercise Rights and Time Off factors. However, Pratt’s importance measures were 0.67 for Exercise Rights and 0.29 for Time Off, so the item was retained in the Exercise Rights factor. Most items loaded most strongly on their corresponding theoretical dimension, with the exceptions of “contract duration” (Q1), “length of employment” (Q2), “exposure to disciplinary action” (Q5), “time off for personal reasons” (Q21) and “time off for family reasons” (Q22). Collectively, the six factors explained 67% of the total variance. Cronbach’s alpha exceeded 0.73 for all factors except Disempowerment (0.54) (Table 5).

Among the physical therapists, all items exhibited factor loadings greater than 0.35. The item “maternity leave” showed cross-loadings on the Rights and Exercise Rights factors. Pratt’s importance measures were 0.782 and 0.213, respectively, supporting its assignment to the Rights factor. Similarly, the item “paid vacation” loaded on both the Rights and Exercise Rights factors, with Pratt’s importance measures of 0.528 and 0.428, respectively, leading to its retention in the Rights factor. Most items loaded most strongly on their corresponding theoretical dimension, with the exceptions of “contract duration” (Q1), “length of employment” (Q2), “net income” (Q12), “time off for personal reasons” (Q21) and “time off for family reasons” (Q22). Together, these six factors explained 71% of the proportional variance. Reliability was acceptable (above 0.70) for all factors (Table 5).

**Table 5:** Factor structure. internal consistency and replicability estimates of the Brazilian version of the *Employment Precariousness Scale* (EPRES-Br). Workers with health conditions reported to the Brazilian Unified National Health System (SUS) in Salvador, and physical therapists in Bahia, Brazil, 2023.

Item	Vulnerability	Wages	Rights	Disempowerment	Exercise Rights	Day off
5A. Workers reported to SUS						
Contract duration			0.650			
Length of employment		0.340				
Definition of schedule				0.639		
Definition of wages				0.913		
Exposure to disciplinary action			-0.352			
Feeling vulnerable	0.752					
Risk of dismissal	0.681					
Authoritarian treatment	0.823					
Feeling replaceable	0.576					
Basic expenses		0.764				
Unforeseen expenses		0.642				
Net income		0.914				
Retirement			0.558			
Severance			0.482			
Maternity leave			0.707			
Unemployment insurance			0.755			
Weekly day off					0.508	
Medical certificate					0.756	
Medical appointment					0.634	0.356
Paid vacation					0.732	
Time off for personal reasons						0.848
Time off for family reasons						0.906
Proportion of variance	0.230	0.140	0.100	0.060	0.080	0.050
Cronbach’s alpha	0.830	0.770	0.730	0.540	0.730	0.950
Latent H-index	0.865	0.878	0.843	0.872	0.851	0.896
Observed H-index	0.762	0.800	0.747	0.720	0.731	0.790
RMSEA	0.000					
CFI	0.999					
TLI	1.018					
5B. Physical Therapists						
Contract duration			0.807			
Length of employment			0.674			
Definition of schedule				0.829		
Definition of wages				0.783		
Exposure to disciplinary action	0.491					
Feeling vulnerable	0.882					
Risk of dismissal	0.602					
Authoritarian treatment	0.846					
Feeling replaceable	0.739					
Basic expenses		0.959				
Unforeseen expenses		0.952				
Net income			0.547			
Retirement			0.802			
Severance			0.465			
Maternity leave			0.773		0.351	
Unemployment insurance			0.714			
Weekly day off					0.543	
Medical certificate					0.795	
Medical appointment					0.586	
Paid vacation			0.504		0.424	
Time off for personal reasons						0.974
Time off for family reasons						0.960
Proportion of variance	0.040	0.080	0.060	0.070	0.180	0.270
Cronbach’s alpha	0.830	0.950	0.830	0.910	0.730	0.980
Latent H-index	0.900	0.975	0.923	0.815	0.876	0.988
Observed H-index	0.919	1.002	0.829	0.707	0.848	1.029
RMSEA	0.099					
CFI	0.953					
TLI	0.905					

Note: Cronbach’s alpha ≥ 0.70 indicates acceptable internal consistency. RMSEA with df = 114 for both samples; 95%CI: 0.0000-0.00331 for workers in Salvador and 0.0635-0.1143 for physical therapists. H-index > 0.80 indicates a well-defined factor. N = 158 for workers in Salvador and N = 399 for physical therapists in Bahia. Factor loadings are presented from |0.30| onwards. Loadings in bold indicate item retention in the factor; non-bold loadings indicate cross-loadings not assigned to a specific factor.

CFI: comparative fit index; RMSEA: root mean square error of approximation; TLI: Tucker-Lewis index.

## Discussion

The EPRES-Br demonstrated good performance in both study samples, with a low proportion of missing data (indicating good acceptability), use of all response categories and satisfactory overall internal consistency reliability across the dimensions of precarious work (Cronbach’s alpha = 0.77 and 0.84, respectively) ^37^. The items exhibited good psychometric properties in the Brazilian context, with moderate (0.40-0.59) or strong (0.60-0.79) item-dimension correlations for nearly all items and moderate to high factor loadings. The six-factor solution was retained in accordance with the original theoretical model, reflecting the consistency of the construct underlying the multidimensional EPRES instrument ^19^. The dimensions of the construct appear conceptually sound; however, our findings suggest that some refinement is warranted, particularly for items 21 and 22 of the Exercise Rights dimension. The moderate to high number of degrees of freedom (df = 114) provides additional support for the reliability of the excellent model fit observed in the Salvador worker sample, reducing the likelihood of RMSEA inflation. A TLI greater than 1 reflects a highly satisfactory model fit based on robust bootstrap estimates of the confidence intervals. Because TLI values may exceed 1, such values are considered indicative of excellent fit and are conventionally interpreted as equivalent to TLI = 1.

### EPRES item performance

Although the EPRES II-2010 includes items that are somewhat more specific to formal employment contracts, we made a few adaptations to reflect the context of high labor informality that characterizes the contemporary world of work. The implications of these adaptations are discussed below.

Both samples yielded the same mean overall EPRES-Br score of 1.62. This value is comparable to those reported in international validation studies: 1.56 for the 10-item EPRES in Central America ^21^, 1.90 for the 23-item Swedish EPRES (EPRES-Se) ^20^, 1.32 for the 21-item Chilean EPRES (EPRES-Ch) ^22^, and 1.70 and 1.60 for the 26-item and 22-item Spanish EPRES versions, respectively ^19^ ^,^ ^25^. In contrast, lower scores were observed in other Spanish subsamples (0.90 in both studies) ^19^ ^,^ ^25^ and in the German validation sample (EPRES-Ge, 0.99; 22 items) ^23^, where standard employment predominated. Thus, the levels of employment precariousness observed in the Brazilian samples were higher than those reported in samples characterized primarily by standard employment ^19^ ^,^ ^20^ ^,^ ^21^ ^,^ ^22^ ^,^ ^23^ ^,^ ^25^. Among these studies, only Vives et al. ^19^ used the 2005 version of the EPRES; all the others used the 2010 version.

Nevertheless, although these international samples have considerable potential for representativeness, direct comparisons should be made with caution because of differences in countries’ economic and legal contexts, sociodemographic profiles and the predominantly non-probability sampling strategies used in validation studies, as discussed by Reichenheim & Bastos ^35^.

With acceptability rates exceeding 90% in both samples, the EPRES-Br performed comparably to previous EPRES validation studies. While most studies reported acceptability rates above 90%, only the Swedish and German studies exceeded 95%. Likewise, the internal consistency reliability observed in this study was comparable to that reported elsewhere: 0.83 in Sweden, 0.83 in Chile, and 0.86 (2010) and 0.82 (2015) in Spain. Reliability was equal to or greater than that reported for Germany (0.77) and Central America (0.50 among informal workers and 0.60 among formal workers) ^19^ ^,^ ^20^ ^,^ ^21^ ^,^ ^22^ ^,^ ^23^ ^,^ ^25^.

The response rate of 44.8% obtained for the Salvador worker sample falls within the range reported in a meta-analysis of remote interview studies, which found an average response rate of 44.1% ^38^.

In the Vulnerability dimension in the Salvador sample, the item “exposure to disciplinary action” showed a below-average item-dimension correlation (Table 2) and a negative loading on the Rights factor, failing to load on the Vulnerability factor corresponding to the original theoretical model (Table 5). This pattern has been reported previously ^25^, with recommendations either to exclude the item from the calculation of the Vulnerability reliability coefficient or to adopt the wording used in the EPRES I version (2005). A Chilean study ^25^ likewise identified the conditional wording of this item as problematic. Its negative phrasing may hinder interpretation, as respondents may construe it as referring to not being subject to retaliation, which is itself a right, thereby explaining its association with the Rights dimension.

The Temporariness dimension exhibited the lowest internal consistency, largely attributable to the item “contract duration”, which partly reflects the weak to moderate correlation between the two items comprising this dimension (Tables 2 and 3). We attribute this finding to limitations in the response options available to self-employed respondents, whose employment arrangements were insufficiently represented in the response categories. This limitation is further reflected in the relatively high proportion of missing responses for this item in both samples (Tables 2 and 3). Similar inconsistencies have been reported in previous studies. Although the Swedish adaptation attempted to address this issue by adding the response options “I do not have a contract” and “I don’t know”, this modification did not result in improved psychometric performance ^20^. Careful wording of questionnaire items to minimize ambiguity is widely regarded as good practice in instrument development ^37^.

### Strengths and limitations of the study

Testing the EPRES-Br to measure employment precariousness in the Brazilian context required including not only formally employed workers but, in particular, those without standard employment status - namely informal workers, individuals on temporary contracts, or involuntary self-employed workers, as in the case of physical therapists subjected to deteriorating working conditions. Therefore, the decision to test the EPRES-Br in the contemporary labor market requires cautious interpretation of the findings, given the prevailing realities. Nevertheless, the results are promising in supporting the scale’s use for measuring employment precariousness in its multidimensionality.

Items from the Temporariness and Rights dimensions loading on the same factor, as well as items related to time off (Q21 and Q22) forming a separate factor distinct from Exercise Rights, revealed certain limitations. Accordingly, we propose revising these items, taking into account their meaning within the contemporary world of work. Based on the accumulated evidence from the various EPRES studies conducted to date, it is desirable to move toward a version that integrates elements from different contractual arrangements and from the diversity of contemporary labor contexts and employment relations.

This study is the first to publish the EPRES II-2010 ^18^ translated into Portuguese and adapted for use in Brazil, and the first to assess the psychometric properties of the EPRES-Br. In addition, the study used two heterogeneous worker samples, both including permanent and temporary workers, ensuring good variability in exposure to employment precariousness.

The use of a flexible EFA approach, based on FACTOR software and framed within structural equation modeling (SEM), made it possible to employ advanced procedures such as RDWLS, RMSEA, CFI and TLI in evaluating the factor structure of the EPRES-Br ^34^.

However, some limitations should be noted. The overall EPRES-Br score was 1.62 on a scale with a maximum score of 4.0, raising questions about its sensitivity to identify more precisely actual levels of exposure to employment precariousness.

We sought to compare the physical therapist sample with another convenience sample study of the same professional group, which showed a similar sociodemographic profile: mean age of 32 years; 79.4% with graduate education; and 67% women ^39^. Therefore, there are no indications of significant differences between respondents and non-respondents. In addition, virtually all potential participants could have received the invitation to participate.

In both samples, latent H-index values were slightly higher than observed H-index values, indicating that the factors are more stable at the theoretical (latent) level than in their empirical operationalization. Nevertheless, the convergence between them is adequate and such differences are common in psychometric data, particularly with ordinal items ^29^.

A methodological choice in this study was the inclusion of social benefits (such as Brazilian Income Transfer Program) in the definition of “net earnings”, under the assumption that such assistance may influence employment precariousness, as it may reduce dependence on more precarious forms of work and thus affect responses to the item ^40^. This adaptation is comparable to the Swedish study, which included tips in net earnings, acknowledging the role of additional income sources to better capture actual income - an approach consistent with ours ^19^ ^,^ ^21^. However, while appropriate, this adaptation may limit direct comparability with studies that considered only “net earnings”.

The EPRES-Br showed adequate psychometric performance in the Brazilian context, with satisfactory reliability and acceptability. Some divergent findings regarding the structure of the original construct are presented in this cross-cultural adaptation study, along with possible explanatory hypotheses.

The psychometric properties of the Vulnerability, Disempowerment and Wages dimensions largely replicate the original scale and support their use, as formulated and translated, for measuring employment precariousness in Brazil. The remaining dimensions (Temporariness, Exercise Rights and Rights) may require item refinement to provide more discriminative measurement aligned with the original construct.

The EPRES aims to operationalize a theoretical construct concerning power relations in employment and at the workplace, capturing both working conditions and perceived risks among workers. In the contemporary context, it is important to emphasize that young people, women, immigrants and racialized workers are disproportionately exposed to the most adverse conditions ^1^.

Nunes et al. ^41^ translated and validated the EPRES proposed by Creed et al. ^42^, designed to measure labor precariousness as “subjective precariousness”, originally applied to university students’ work experiences. The EPRES, in contrast, focuses on power relations in employment and at the workplace, capturing contractual aspects, working conditions and labor relations ^6^. Moreover, its validation history across 11 countries supports confidence in its applicability and adaptability across contexts.

The administration of the EPRES-Br to workers with different types of employment relationships, including informal labor market participation, more closely reflects contemporary labor realities and extends beyond standard wage employment. This broader scope may influence how certain dimensions contribute to the measurement of precariousness and should be interpreted with the caution required in adaptation studies.

It is essential that the multidimensional construct of employment precariousness continues to be measured in epidemiological studies to generate stronger evidence on the social determination of health and illness among workers. The increasing erosion of working conditions in the contemporary labor world requires sustained attention to precarious work and its constituent dimensions, in order to better explain it and to support public policies aimed at regulating corporate labor practices and identifying ambiguous employment relations ^3^.

## Data Availability

The research data are not available.
