# ERRATUM: Measurement of precarious work in Brazil: translation, adaptation and dimensionality of the *Employment Precariousness Scale* (EPRES-Br).

**DOI:** 10.1590/0102-311XEREN206125

**Published:** 2026-11-09

**Santana VR, Vives-Vergara A, Jesus GRS, Fernandes RCP. Measurement of precarious work in Brazil: translation, adaptation and dimensionality of the *Employment Precariousness Scale* (EPRES-Br). Cad Saúde Pública 2026; 42:e00206125.**

Where it reads:

Victor Rocha Santana ^1^

Alejandra Vives-Vergara ^2^

Gláucio Roberto Santana de Jesus ^3^

Rita de Cássia Pereira Fernandes ^3^

^1^ Instituto de Saúde Coletiva, Universidade Federal da Bahia, Salvador, Brasil.

^2^ Universidad Católica de Chile, Santiago, Chile.

^3^ Programa de Pós-graduação em Saúde, Ambiente e Trabalho, Universidade Federal da Bahia, Salvador, Brasil.

Correspondence

V. R. Santana

Instituto de Saúde Coletiva, Universidade Federal da Bahia.

Rua Basílio da Gama s/n, Campus Universitário Canela, Salvador, BA 40110-040, Brasil.

vilarongavictor@gmail.com

It should read:

Victor Rocha Santana ^1^

Alejandra Vives-Vergara ^2^

Gláucio Roberto Santana de Jesus ^3^

Rita de Cássia Pereira Fernandes ^1,3^

^1^ Programa de Pós-graduação em Saúde Coletiva, Universidade Federal da Bahia, Salvador, Brasil.

^2^ Escuela de Salud Pública, Pontificia Universidad Católica de Chile, Santiago, Chile

^3^ Programa de Pós-graduação em Saúde, Ambiente e Trabalho, Universidade Federal da Bahia, Salvador, Brasil.

*Correspondence

V. R. Santana

Programa de Pós-graduação em Saúde Coletiva, Universidade Federal da Bahia.

Rua Basílio da Gama s/n, Campus Universitário Canela, Salvador, BA 40110-040, Brasil.

vilarongavictor@gmail.com

Where it reads:

Contributors

V. R. Santana contributed with the study conception, data analysis and interpretation, and writing; and approved the final version. A. Vives-Vergara contributed with the study conception, writing, and review; and approved the final version. G. R. S. Jesus contributed with the study conception, writing, and review; and approved the final version. R. C. P. Fernandes contributed with the study conception, writing, and review; and approved the final version.

It should read:

Contributors

V. R. Santana contributed with the study conception, data collection, analysis and interpretation, and writing; and approved the final version. A. Vives-Vergara contributed with the writing and review; and approved the final version. G. R. S. Jesus contributed with the data collection and review; and approved the final version. R. C. P. Fernandes contributed with the study conception, data analysis and interpretation, writing, and review; and approved the final version.

Responsible for approval:

Editor-in-Chief Marilia Sá Carvalho (0000-0002-9566-0284)

Editor-in-Chief Luciana Dias de Lima (0000-0002-0640-8387)

Editor-in-Chief Luciana Correia Alves (0000-0002-8598-4875)

