# Supplementary material for: Measurement of precarious work in Brazil: translation, adaptation and dimensionality of the Employment Precariousness Scale (EPRES-Br)
Source: Cad Saude Publica. 2026 Aug 21;42:e00206125. doi: 10.1590/0102-311XEN206125 (PMC13505639; doi:10.1590/0102-311XEN206125)
Supplement: Supplementary file 1 [file 1678-4464-csp-42-EN206125-s.pdf]

Suplemento de artigo Mensuração do trabalho precário no Brasil: tradução, adaptação e dimensionalidade da Escala de Precariedade do Emprego (EPRES-Br)

EPRES II – 2010 revisado
Referência Vives A. Employment Precariousness Scale II - 2010 Basic Pack. Departamento de Salud Pública Pontificia Universidad Católica de Chile;2021.
DIMENSION / ITENS
TEMPORARINESS
1 - The duration of your contract is
0 = Permanent contract 1 = 1 year or more 2 = [...] 3[...] 4[...]
2- For how long (in all) have you been working for this company?
0 = More than 10 years 1 = Between 5 and 10 years 2 = [...] 3 [...] 4 [...]
DISEMPOWEMENT
How did you settle the following employment conditions?
3- Workplace schedule
4-Wages or salary
0 = By collective bargaining/agreement 1 = In my work team 2 [...]3 [...] 4[...]
VULNERABILITY
5- you are able to demand better working conditions without being exposed to reprisals?
0 = Always 1 = Many times 2 [...] 3[...] 4 [...]
6- you are defenseless towards unfair treatment by your superiors
7- you would be fired for not doing all what you are asked to do
8- you are treated in an authoritarian manner
9- you are made to feel you can be easily replaced
0 = Never 1 = Rarely 2 [...] 3 [...] 4 [...]
WAGES
10- Does your current salary allow you to cover your basic needs?
11- Does your current salary allow you to cover unexpected expenses?
0 = Always 1 = Many times 2 [...] 3 [..] 4 [...]
12- How much is your take home (net) monthly wage or salary?
0 => 2400 euros 1 => 1500-2400 Euros 2 [...] 3 [...] 4 [...]
RIGHTS
Of the following benefits, which do you have a right to...
13- Pension for old age or disability
14- Severance pay
15- Maternity / paternity leave
16- Unemployment benefit / compensation
0 = Yes 1 = No 2 [...]
EXERCISE RIGHTS
Indicate how often you can exercise the following rights without obstacles...
17- Weekly holidays
18- Take sick leave
19- Go to the doctor
20- Take vacations
21- Request a day off for personal reasons when needed
22- Request a day off for family affairs when needed
0 = Always 1 = Many times 2 [...] 3 [...] 4 [...]

EPRES – BR
DIMENSÃO / ÍTENS
TEMPO DE PERMANÊNCIA
1. A duração do seu contrato é:
0= Contrato permanente (carteira de trabalho assinada (CLT), concursado) 1= Temporário, um ano ou mais 2= [...] 3= [...] 4= [...]
2. Há quanto tempo (ao todo) você trabalha nesta firma/empresa?
0= Mais de 10 anos 1= De 5 a 10 anos 2= [..] 3= [...] 4= [...]
DESEMPODERAMENTO
3. Horário de trabalho
4. Remuneração ou salário
0= Por acordo coletivo / negociação 1=Na minha equipe de trabalho 2= [...] 3= [...] 4[...]
VULNERABILIDADE
5. Você é capaz de pedir por melhores condições de trabalho sem ser exposto a punições?
0= Nunca 1= Raramente 2= [...] 3= [...] 4= [...]
6. Com que frequência você se sente fragilizado diante do tratamento injusto de seus superiores?
7.Você seria demitido por não fazer tudo que lhe pedem?
8. Você é tratado de forma autoritária?
9. Você é levado a sentir que pode ser facilmente substituído?
0= Nunca 1= Raramente 2= [...] 3= [...] 4= [...]
REMUNERAÇÃO
10. Seu salário/remuneração atual te permite cobrir as suas necessidades básicas?
11. Seu salário/ remuneração atual te permite cobrir despesas inesperadas?
0= Sempre 1= Muitas vezes 2= [...] 3= [...] 4= [...]
12. Somando salário/remuneração e benefícios sociais, qual é o seu ganho líquido mensal?
0= Mais que 10 salários mínimos 1= Entre 6,5 e 10 salários mínimos 2= [...] 3= [...] 4= [...]
DIREITOS
Dos seguintes benefícios, você tem direito à:
13. Aposentadoria por idade ou invalidez?
14. Indenização?
15. Licença maternidade/paternidade?
16. Seguro-desemprego?
0= Sim 1= [...] 2= [...]
EXERCÍCIO DOS DIREITOS
Indique com que frequência você exerce os Seguintes direitos sem dificuldades:
17. Descanso semanal
18. Licença médica
19. Ir ao médico (para consultas)
20. Férias
21. Solicitar folga por motivos pessoais quando necessário
22. Solicitar folga por motivos familiares quando necessário
0= Sempre 1= Muitas vezes 2= [...] 3= [...] 4= [...]

Nota: esta lista de itens do EPRES para as aplicações do questionário tem finalidade meramente ilustrativa. Atualmente, já estão disponíveis versões validadas do questionário em espanhol, inglês, grego, alemão e sueco. Informações adicionais sobre o EPRES-Br podem ser solicitadas aos autores deste manuscrito.
