# Supplementary material for: Measurement of precarious work in Brazil: translation, adaptation and dimensionality of the Employment Precariousness Scale (EPRES-Br)
Source: Cad Saude Publica. 2026 Aug 21;42:e00206125. doi: 10.1590/0102-311XEN206125 (PMC13505639; doi:10.1590/0102-311XEN206125)
Supplement: Translation

# Translation: Mensuração do trabalho precário no Brasil: tradução, adaptação e dimensionalidade da *Escala de Precariedade do Emprego* (EPRES-Br)

**DOI:** 10.1590/0102-311XPT206125

**Authors:** Victor Rocha Santana, Alejandra Vives-Vergara, Gláucio Roberto Santana de Jesus, Rita de Cássia Pereira Fernandes

**Affiliations:** 1 Instituto de Saúde Coletiva, Universidade Federal da Bahia, Salvador, Brasil.; 2 Universidad Católica de Chile, Santiago, Chile.; 3 Programa de Pós-graduação em Saúde, Ambiente e Trabalho, Universidade Federal da Bahia, Salvador, Brasil.

**Keywords:** Emprego Precário, Psicometria, Vigilância em Saúde do Trabalhador, Estudo de Validação, Epidemiologia e Bioestatística

## Abstract

Este estudo analisou as propriedades psicométricas - aceitabilidade, confiabilidade e estrutura fatorial - da versão brasileira da *Escala de Precariedade no Emprego* (EPRES-Br), aplicada em duas amostras de trabalhadores brasileiros: trabalhadores com agravos de notificação compulsória pelo Sistema Único de Saúde em Salvador e fisioterapeutas na Bahia. Ao todo, 638 indivíduos com vínculos formais e informais de trabalho responderam ao instrumento. A aceitabilidade, a consistência interna e a dimensionalidade da escala foram avaliadas por meio da estatística descritiva e analítica, incluindo análise fatorial com metodologia para variáveis categóricas, adequada a dados ordinais e baseada em correlações policóricas. A EPRES-Br apresentou alta aceitabilidade (acima de 90%) e consistência interna aceitável (alfa de Cronbach = 0,77 para trabalhadores de Salvador e 0,84 para fisioterapeutas). As dimensões Remuneração, Desempoderamento e Vulnerabilidade confirmaram o construto teórico, já Tempo de Permanência e Direitos carregaram no mesmo fator, e dois itens sobre folgas carregaram fora da dimensão teórica original (Exercício dos Direitos). Conclui-se que a EPRES-Br apresenta boas propriedades psicométricas para uso em estudos epidemiológicos no Brasil, embora sejam necessárias revisões de alguns itens, considerando o significado deles para o contexto do mundo do trabalho.

## Introdução

O trabalho precário é um determinante social de saúde, com evidências de sua associação com a saúde mental, distúrbios musculoesqueléticos e acidentes ^1^ ^,^ ^2^. Ademais, o trabalho precário atinge relações informais e formais de trabalho, levando à incerteza, insegurança, relações ambíguas, restrição de direitos, baixa remuneração e restrição à representação coletiva de direitos trabalhistas ^3^. Situações, portanto, de vulnerabilização social.

Apesar do interesse em abordar o trabalho precário, modelos teóricos acerca de sua multidimensionalidade são bem recentes ^2^ ^,^ ^4^. Da mesma forma, a operacionalização de um instrumento de mensuração capaz de abranger essa multidimensionalidade vem ocorrendo há pouco mais de uma década, a partir de desenvolvimentos na Espanha, com subsequentes estudos de validação de instrumento de medida ^5^ ^,^ ^6^ ^,^ ^7^ ^,^ ^8^ ^,^ ^9^.

A história do trabalho precário suplantando o emprego padrão em países industrializados ocidentais é conhecida ^1^. No entanto, no Brasil, a universalização das relações de assalariamento com garantia de direitos, carga horária integral e negociação coletiva não chegou a ser consolidada, haja vista a marcada exclusão sistemática de mulheres e negros mediante excedente estrutural da força de trabalho, trabalho análogo à escravidão e trabalho não remunerado (especialmente na economia do cuidado) ^10^ ^,^ ^11^. Portanto, as modalidades mais recentes de trabalho precário ^12^ se agregam às demais formas já existentes de precarização, não superadas.

O Brasil, sendo considerado um país de rendimento médio superior pelo Banco Mundial, figura próximo à 8ª economia do mundo ^13^. Em abril de 2024, a taxa de desocupação dessazonalizada registrou 7,5%, declinante, com uma população ocupada de 100,8 milhões de pessoas, em um país com 203 milhões de habitantes. Daqueles ocupados em 2023, havia 39,4 milhões de trabalhadores informais (ou 39,2%) ^14^. No entanto, apesar do nível recorde de formalização de contratos de trabalho nos últimos 12 anos, a duração daqueles empregos formais cessa em 24 meses para 50% ^15^.

A crise sanitária e econômica da pandemia de COVID-19 aprofundou essas contradições internas. Como consequência, a ampliação das vulnerabilidades dos trabalhadores se materializa na geração de pobreza e de trabalho precário, em retroalimentação mútua ^1^ ^,^ ^16^.

Embora a definição de trabalho precário careça ainda de maior consenso, podem ser referidas definições correntes que apontam para o trabalho inseguro, no sentido de sua permanência, com renda insuficiente e desprovido de proteção social e trabalhista ^4^.

Estudos que investigaram a associação de trabalho precário com desfechos negativos em saúde mental demonstraram associação positiva, especialmente utilizando exposições multidimensionais. Estudos com exposições unidimensionais tais como insegurança percebida no emprego e emprego temporário apresentavam baixa sensibilidade, sendo desencorajados em pesquisas futuras ^17^.

A *Escala de Precariedade do Emprego* (EPRES) de Amable ^5^, usada para mensurar a precariedade do emprego assalariado em sua multidimensionalidade em estudos epidemiológicos, tem seis dimensões, com 22 itens ^18^. Sobre ela, foram identificados sete estudos que investigaram suas propriedades psicométricas, conduzidos em cinco países (Espanha, Suécia, Chile, Grécia e Alemanha) e em uma região com seis países da América Central ^19^ ^,^ ^20^ ^,^ ^21^ ^,^ ^22^ ^,^ ^23^ ^,^ ^24^. Depois de um primeiro estudo de validação com 26 itens ^19^, a Espanha fez nova investigação em 2015 ^25^ com a segunda versão da EPRES de 2010 (EPRES II) e 22 itens.

No geral, esses estudos de validação corroboraram a estrutura teórica da EPRES, confirmando as seis dimensões. A exceção foi o estudo centro-americano que aplicou e analisou questões de apenas quatro dimensões, ainda assim chegando a resultados convergentes com os dos demais.

Não há publicações de estudos no Brasil utilizando a EPRES e nem sua tradução para o português e o contexto brasileiro, apesar de ser um país com alta precarização e significativo adoecimento relacionado ao trabalho ^2^. Com isso, ocorrem dificuldades de dimensionar a população de trabalhadores expostos ao trabalho precário, limitando novas políticas públicas. Assim, um instrumento para mensurar trabalho precário deve colaborar com a maior convergência conceitual e metodológica para permitir comparações entre países.

Nesse sentido, utilizamos a EPRES em duas pesquisas realizadas em 2023. O instrumento foi traduzido para o português brasileiro (EPRES-Br) e feita adaptação transcultural para o contexto do nosso mercado de trabalho. Sendo esse marcado por elevada heterogeneidade e informalidade, e considerando discussões de autores da escala, explora-se a aplicação do instrumento também em trabalhadores com diferentes modalidades de inserção laboral, avaliando em que medida sua estrutura teórica se mantém válida nesse contexto ^26^. O objetivo principal deste estudo foi avaliar a aceitabilidade, a confiabilidade e a dimensionalidade da EPRES-Br. Como objetivos secundários, pretendemos caracterizar as amostras e descrever o processo de tradução e adaptação do instrumento, bem como o desempenho dos fatores correspondentes às dimensões do construto teórico original. Ademais, admitia-se que os itens da dimensão Tempo de Permanência poderiam mostrar piores propriedades psicométricas em um contexto de maior informalidade, com base no acúmulo de evidências dos estudos de validação já conduzidos.

## Métodos

### Tradução e adaptação da EPRES-Br

(1) Para se alcançar os objetivos do estudo, a EPRES II-2010 ^18^ foi traduzida do Inglês (versão fornecida pelo Grupo de Pesquisa em Desigualdades em Saúde e Ecologia/Rede de Condições de Emprego [GREDS-EMCONET] da Universidade Pompeu Fabra; Espanha, https://www.upf.edu/web/greds-emconet/) para o Português do Brasil e realizada a adaptação transcultural e para o mercado de trabalho brasileiro, conforme descrito a seguir.

(2) Na questão referente à remuneração, definida originalmente por ganho líquido mensal, decidiu-se orientar os participantes a considerar, além da renda auferida pelo trabalho, benefícios sociais recebidos de maneira continuada, como o Programa Bolsa Família. Essa adaptação foi adotada para refletir as condições socioeconômicas objetivas de trabalhadores em contextos de maior vulnerabilidade, nos quais diferentes fontes de rendimento podem compor os recursos disponíveis para a reprodução social.

(3) A tradução foi realizada no grupo de pesquisa Epidemiologia, Vigilância em Saúde, Estudo do Trabalho (Conselho Nacional de Desenvolvimento Científico e Tecnológico - CNPq) por três pesquisadores: uma epidemiologista e dois pós-graduandos da Universidade Federal da Bahia (UFBA), bilíngues. Esses coautores do presente artigo são profissionais de saúde da Bahia e o grupo acumula longa trajetória em pesquisa epidemiológica no mundo do trabalho, nos contextos baiano e brasileiro.

(4) A retrotradução da versão em Português para o Inglês foi realizada por professor de Língua Inglesa, nativo da Inglaterra e fluente em Português.

(5) Na sequência, três pesquisadores independentes, um epidemiologista e dois cientistas sociais em Saúde do Trabalhador, fizeram a verificação das versões produzidas: a tradução para o Português do Brasil e o texto da retrotradução.

(6) Concluída essa etapa, ainda com o fim de contribuir com a adequação da linguagem, o instrumento foi aplicado a trabalhadoras associadas ao Sindicato de Trabalhadoras Domésticas da Bahia, sendo informais e formais. A escolha dessas trabalhadoras foi determinada pelo acesso dos pesquisadores a essa entidade sindical. Ao final, foi requisitado um retorno livre acerca da experiência de cada respondente. Apesar do número pequeno de participantes, os resultados foram avaliados como úteis, dado que o entendimento delas sobre o sentido das questões e opções de resposta foi satisfatório.

### Sujeitos e desenho de estudo

A pesquisa com trabalhadores com agravos de notificação pelo Sistema Único de Saúde (SUS) objetivou testar a hipótese de associação entre trabalho precário e violência autoprovocada (conceito que engloba ideação suicida, comportamento suicida e automutilação) ^27^. Este foi um estudo de caso-controle cujos resultados estão submetidos para publicação. Os participantes foram selecionados a partir do Sistema de Informação de Agravos de Notificação (SINAN), por meio de notificações por agravos de violência autoprovocada (grupo caso) e por atendimento antirrábico humano (grupo controle). Os critérios de inclusão foram auferimento de remuneração pelo próprio trabalho, além do número de telefone acessível e ser maior de idade. Já os critérios de exclusão foram óbito, ser estudante ou aposentado. Todos os possíveis casos com número de telefone disponível foram incluídos para contacto, enquanto os possíveis controles foram sorteados numa proporção de 2 para 1 caso, em uma amostragem aleatória simples. Os indivíduos foram entrevistados por telefone por entrevistadores treinados e aprovados para a pesquisa, totalizando 180 entrevistados de um grupo de 402 elegíveis, perfazendo uma proporção de resposta de 44,8%.

A pesquisa com fisioterapeutas do Estado da Bahia investigou os fatores associados a trabalho precário nesta categoria profissional. Este foi um estudo de corte transversal, por meio de *websurvey*, cujos resultados também estão submetidos para publicação. Os critérios de inclusão foram ser fisioterapeuta em atividade no Estado da Bahia, auferindo renda por seus serviços. Foram obtidos 458 questionários completos.

Conforme a natureza de uma pesquisa online, diferentes meios permitiram a disseminação do *link* do questionário, seja pela equipe de pesquisa ou por fisioterapeutas coordenadores de serviços, que foram solicitados a compartilhá-lo em grupos de trabalho e contatos individuais no Whatsapp, com a solicitação de compartilhamento do *link* para colegas fisioterapeutas. Portanto, não é possível mensurar quantos fisioterapeutas receberam o *link*, nem calcular a proporção de resposta, dada esta limitação em pesquisa online. Embora não seja possível garantir que todos os trabalhadores tenham sido informados sobre esta pesquisa, as estratégias híbridas de divulgação permitiram um alcance razoável da categoria de fisioterapeutas, já que todos os profissionais têm acesso à internet. Nesse sentido, a não participação no estudo não parece ter uma razão específica, ou seja, não há elementos que apontem diferenças relevantes entre os respondentes e não respondentes. Entretanto, o uso de amostragem de conveniência deve ser sempre considerado uma limitação potencial, dado não ser possível assegurar a representatividade da amostra.

Desta forma, a população total deste estudo, em duas amostras, é de 638 trabalhadores. A coleta de dados das duas pesquisas ocorreu em 2023, como parte de dissertação de mestrado em Saúde, Ambiente e Trabalho e de tese de doutorado em Saúde Coletiva. Não é possível descartar algum viés de seleção, dadas as dificuldades de acesso aos casos - que apresentaram um desfecho extremo de saúde, violência autoprovocada no estudo com população de Salvador - e da natureza online do inquérito com os fisioterapeutas.

### Abordagem estatística dos dados

A EPRES-Br testou os 22 itens em seis dimensões da EPRES II-2010 revisada, a saber: Tempo de Permanência (2 itens), Desempoderamento (2 itens), Vulnerabilidade (5 itens), Remuneração (3 itens), Direitos (4 itens) e Exercício dos Direitos (6 itens). A escala de resposta também seguiu o instrumento original, com cinco itens ordinais crescentes para todas as dimensões, com exceção da dimensão Direitos, com três itens ordinais crescentes ^18^.

Conforme previsto pelos autores da escala original, os subescores das seis dimensões foram definidos pela média dos valores das respostas aos itens respectivos, formando uma escala de 0 a 4. Já o escore global foi uma média das seis dimensões, podendo abranger escores de 0 a 4, sendo zero o menor nível possível de precariedade ^18^.

Na primeira abordagem estatística dos dados, foram descritos os itens da escala EPRES-Br por meio da média, desvio padrão, com registro de dados perdidos. Em seguida, realizou-se com os itens de cada dimensão da escala a correlação item-dimensão de Spearman, eliminando o item de interesse para evitar sobreposição. Além disso, foram registrados efeitos piso e teto, ou seja, a proporção de respondentes com o mais baixo escore e o mais alto escore possível, e obtido o coeficiente alfa de Cronbach, como um indicador de confiabilidade da consistência interna ^28^. Não foi realizada a imputação de dados faltantes. E quando aplicável, o número de observações válidas (n) foi apresentado por item ou dimensão. Eventuais variações ocorreram por dados faltantes.

A análise fatorial com metodologia para variáveis categóricas (CVM-FA; *categorical variable methodology factor analysis*) foi realizada com cada uma das amostras das pesquisas descritas, para se explorar a estrutura fatorial da EPRES-Br ^29^. A análise foi feita utilizando-se uma matriz de correlação policórica e o método de extração de fatores de mínimos quadrados ponderados diagonalmente e robustos (RDWLS; *robust diagonally wighted least squares*) ^28^. O número de fatores a ser retido baseou-se no modelo teórico original, que orientou a análise paralela com 500 amostras aleatórias em *bootstrap* ^18^ ^,^ ^30^, e a rotação empregada foi a *robust promin* ^31^. A técnica de análise paralela foi utilizada em um dos estudos prévios de validação da EPRES ^20^. Para essa etapa, excluímos respondentes com pelo menos um *missing*, adotando a técnica dos dados completos.

A adequação dos modelos foi avaliada por meio dos índices de ajuste de bondade erro quadrático médio de aproximação (RMSEA; *root mean square error of approximation*), índice de ajuste comparativo (CFI) e índice de Tucker-Lewis (TLI). Utilizou-se como parâmetro para RMSEA valores menores que 0,08 e valores de CFI e TLI acima de 0,90 ^32^.

A estabilidade dos modelos foi avaliada mediante índice H ^29^. Esse índice permite avaliar em que medida alguns itens representam um fator comum. Seus valores vão de 0 a 1. Quando ocorrem valores maiores que 0,80, indica-se uma variável latente bem definida, que possivelmente seja mais replicável em outros estudos. Já valores baixos indicam uma variável latente mal definida e geralmente menos replicável em outros estudos. Também empregou-se a medida de importância de Pratt ^33^, de modo a interpretar alguma divergência entre cargas fatoriais cruzadas, quando estas tiveram carga igual ou superior a |0,3|. Essa medida informa a proporção que cada fator explica um item. A confiabilidade foi apresentada com alfa de Cronbach, de modo que valores iguais ou acima de 0,70 foram considerados aceitáveis.

Usou-se o software Stata, versão 14.2 (https://www.stata.com), para a abordagem descritiva e o FACTOR, versão 12.04.05 (https://psico.fcep.urv.cat/utilitats/factor/index.html), para a análise fatorial exploratória ^34^. O FACTOR é um software de acesso livre e de uso orientado para análise fatorial exploratória (EFA, CVM-FA) ou semiconfirmatória (SCFA).

Os sujeitos concordaram em participar por intermédio de Termo de Consentimento Livre e Esclarecido. Os dados obtidos foram anonimizados e as pesquisas receberam autorização do Comitê de Ética em Pesquisa da Faculdade de Medicina da Bahia/UFBA (registros nº 5.258.142 e nº 6.046.587).

## Resultados

### Tradução e adaptação da EPRES-Br

A etapa de tradução e adaptação da EPRES II-2010 para o contexto brasileiro exigiu as seguintes definições: na Q1, “a duração do seu contrato é”, a categoria de resposta “contrato permanente” foi acrescida de termos explicativos adequados ao contexto brasileiro - Carteira de Trabalho assinada e concurso público, entre parênteses. Na dimensão Remuneração (questões 10 a 12) foi acrescido o termo “remuneração”, como sinônimo ampliado, adotando-se “Seu salário (ou remuneração)”. Isso se deu para contemplar aqueles respondentes que tinham ganhos pelo trabalho, mas não recebiam salário. Na Q12, sobre ganho líquido, foi apontado que esse montante deveria compreender salário (ou remuneração) e benefícios (Bolsa Família) ^35^. Além disso, foram convertidos os valores em Euros, da escala original em inglês, para salário mínimo do Brasil em Reais com base em valor de 2022 (Material Suplementar; https://cadernos.ensp.fiocruz.br/static//arquivo/suppl-e00206125_3895.pdf).

### Sujeitos de estudo

Entre os participantes, predominaram mulheres, raça/cor não branca e expostos à precarização de moderada a alta (EPRES-Br > 1,3 para trabalhadores de Salvador e > 1,29 para fisioterapeutas). Os trabalhadores notificados pelo SUS têm menor escolaridade. Já os fisioterapeutas, nível universitário. Mais da metade dos trabalhadores de Salvador e dos fisioterapeutas tinham contratos permanentes (54,8% e 59,9%, respectivamente). Ainda assim, houve uma expressiva participação de trabalho informal ou contratos temporários. A permanência na empresa, no geral, foi de menos de 10 anos para 70% dos trabalhadores, sendo mais curta nas modalidades de contrato temporário (Tabela 1).

**Tabela 1:** Características sociodemográficas e ocupacionais das populações de trabalhadores de Salvador e fisioterapeutas da Bahia. Brasil, 2023.

Características	Trabalhadores de Salvador (N = 180)		Fisioterapeutas da Bahia (N = 458)	
	n	%	n	%
Sexo				
Feminino	114	63,3	330	72,0
Masculino	66	36,7	128	28,0
Raça/Cor da pele				
Amarela, branca	26	14,4	140	31,0
Indígena, parda, preta	154	85,6	316	69,0
Escolaridade *				
Mais escolarizados	30	16,7	405	88,4
Menos escolarizados	150	83,3	53	11,6
Situação conjugal e filhos				
Solteiro com filhos/Casado/Vive junto	118	65,6	269	58,7
Solteiro	62	34,4	189	41,3
Modalidade contratual **				
Trabalho padrão	120	67,0	356	77,9
Vínculo precário	59	33,0	101	22,1
Tempo no emprego atual (anos)				
≥ 5	89	49,7	236	54,5
< 5	90	50,3	197	45,5

Nota: o número de observações válidas pode variar entre variáveis em função de dados faltantes.

* Escolaridade (dicotomizada): trabalhadores de Salvador - mais escolarizados: Ensino Médio completo ou Superior; menos escolarizados: de analfabetismo a Ensino Médio incompleto; fisioterapeutas - mais escolarizados: especialização, mestrado ou doutorado; menos escolarizados: graduação;

** Modalidade contratual (dicotomizada): trabalho padrão (trabalhadores de Salvador): emprego com carteira assinada ou trabalho autônomo voluntário; vínculo precário (trabalhadores de Salvador): emprego sem carteira assinada ou trabalho autônomo involuntário; trabalho padrão (fisioterapeutas): servidor público, carteira assinada, empresário ou pessoa jurídica voluntária; vínculo precário (fisioterapeutas): contrato temporário ou pessoa jurídica involuntária.

As ocupações na amostra de trabalhadores notificados pelo SUS são do setor da saúde (15,2%), comércio (6,3%), serviços de limpeza (5%), transporte (5%) e trabalho doméstico (5,6%), dentre outros menos representados.

A amplitude da idade foi de 18 a 77 anos para os trabalhadores notificados pelo SUS, com mediana de 39,5 anos, e para os fisioterapeutas foi de 23 a 70 anos, com mediana de 37 anos.

### Propriedades psicométricas da EPRES-Br

A Tabela 2 mostra a proporção de dados perdidos, média, desvio padrão, frequências de respostas e correlação item-dimensão por item. Para os trabalhadores de Salvador, houve alta proporção de respostas (acima de 90%), com a utilização de todas as opções de respostas. Os itens se correlacionaram mais com a dimensão correspondente do que com outras dimensões, com exceção de Q1, Q5 e Q13 (Tabela 2).

**Tabela 2:** Estatística descritiva de itens versão brasileira da *Escala de Precariedade no Emprego* (EPRES-Br) e correlação item-dimensão de Spearman. Trabalhadores com agravos notificados pelo Sistema Único de Saúde (SUS) de Salvador, Bahia, Brasil, 2023.

Dimensão/Item	Dados perdidos (%)	Média	DP	Frequência de valores de resposta (%)					Correlação item-dimensão de Spearman					
				0	1	2	3	4	Tempo de Permanência	Desempoderamento	Vulnerabilidade	Remuneração	Direitos	Exercício dos Direitos
Tempo de Permanência														
Duração do contrato	7,78	0,90	1,11	50,6	8,3	27,7	2,8	2,8	0,26	0,06	-0,09	0,15	0,48	0,24
Tempo trabalhado	0,56	1,64	1,40	28,9	20,6	21,1	15,0	13,8	0,26	0,15	0,04	0,16	0,19	0,11
Desempoderamento														
Quem define horário	1,67	2,04	1,54	20,0	6,7	28,3	36,1	7,2	0,15	0,59	-0,03	0,01	0,07	0,14
Quem define remuneração	1,67	2,18	1,33	21,7	2,8	21,7	41,1	11,0	0,12	0,59	-0,03	-0,03	0,16	0,18
Vulnerabilidade														
Exposto a punições	1,11	1,96	1,64	33,3	6,7	17,8	12,8	28,3	0,00	-0,03	0,08	0,19	-0,07	0,11
Sente-se fragilizado	0,00	1,00	1,21	48,3	20,6	20,0	4,4	6,7	0,00	-0,02	0,50	0,33	0,07	0,16
Risco de demissão	1,67	0,94	1,35	55,0	19,4	10,0	2,2	11,7	0,02	-0,02	0,47	0,18	0,05	0,17
Tratamento autoritário	1,11	0,83	1,18	59,4	11,7	18,4	4,4	5,0	0,00	-0,02	0,54	0,34	0,15	0,23
Sentir-se substituível	1,67	1,66	1,52	32,8	16,1	23,9	3,3	22,2	-0,09	0,02	0,34	0,05	-0,08	0,08
Remuneração														
Despesas básicas	0,00	1,63	1,43	34,4	10,0	26,7	15,6	13,3	0,14	0,02	0,19	0,70	0,21	0,25
Despesas inesperadas	0,56	2,53	1,31	11,0	8,9	25,6	23,9	30,0	0,09	0,01	0,31	0,65	0,21	0,22
Ganho líquido	1,11	3,19	1,03	1,1	6,7	17,8	20,6	52,7	0,36	0,02	0,25	0,55	0,28	0,11
Direitos														
Aposentadoria	1,11	0,58	0,71	54,4	31,7	12,8	NA	NA	0,40	0,16	0,04	0,26	0,40	0,11
Indenização	1,67	0,85	0,73	35,0	43,3	20,0	NA	NA	0,13	0,11	-0,02	0,13	0,42	0,14
Licença-Maternidade	1,67	0,51	0,62	55,0	36,7	6,6	NA	NA	0,40	0,09	0,05	0,26	0,48	0,28
Seguro	1,11	0,66	0,58	38,9	54,4	5,6	NA	NA	0,18	-0,02	-0,06	0,06	0,44	0,20
Exercício de Direitos														
Folga semanal	1,11	0,86	1,28	63,3	6,1	15,0	8,9	5,6	0,11	0,09	0,13	0,17	0,13	0,47
Atestado médico	1,11	1,43	1,57	49,4	2,8	15,6	17,2	13,9	0,10	0,11	0,19	0,20	0,23	0,56
Consulta médica	1,11	1,01	1,25	55,0	7,2	18,9	16,1	1,7	0,20	0,17	0,15	0,18	0,14	0,72
Férias	1,11	1,36	1,64	54,4	2,8	11,7	11,7	18,3	0,30	0,18	0,03	0,08	0,38	0,60
Folga por motivos pessoais	1,67	1,69	1,48	36,1	5,6	22,2	21,7	12,7	0,09	0,03	0,17	0,19	0,04	0,40
Folga por motivos familiares	1,67	1,76	1,53	36,7	3,9	20,0	22,2	15,5	0,18	0,17	0,14	0,20	0,12	0,59

Nota: correlações destacadas em negrito evidenciando cada conjunto de itens do respectivo construto teórico; n por item pode variar em função dados perdidos (*missing*).

DP: desvio padrão; NA: não se aplica.

Entre os fisioterapeutas (Tabela 3), também houve alta proporção de respostas (> 90%) e todas as opções em cada item foram usadas pelos respondentes. A correlação, em geral, foi maior com a dimensão correspondente do que com outras, com exceção de Q1 (duração de contrato), Q12 (ganho líquido), Q15 (licença maternidade) e Q19 (férias) (Tabela 3).

**Tabela 3:** Estatística descritiva de itens da da versão brasileira da *Escala de Precariedade no Emprego* (EPRES-Br) e correlação item-dimensão de Spearman. Fisioterapeutas da Bahia, Brasil, 2023.

Dimensão/Item	Dados perdidos (%)	Média	DP	Frequência de valores de resposta (%)					Correlação item-dimensão de Spearman					
				0	1	2	3	4	Tempo de Permanência	Desempoderamento	Vulnerabilidade	Remuneração	Direitos	Exercício dos Direitos
Tempo de Permanência														
Duração do contrato	8,08	0,62	0,94	59,9	11,2	18,2	1,3	1,3	0,42	1,14	-0,07	0,09	0,57	0,22
Tempo trabalhado	5,46	1,50	1,22	23,5	27,9	22,5	13,6	7,0	0,42	0,13	0,06	0,30	0,37	0,20
Desempoderamento														
Quem define horário	5,90	1,88	1,24	19,8	13,8	22,7	33,2	4,6	0,11	0,55	0,08	0,12	0,21	0,13
Quem define remuneração	6,11	2,03	1,39	26,8	3,2	8,7	50,4	4,8	0,19	0,55	0,09	0,14	0,24	0,16
Vulnerabilidade														
Exposto a punições	5,02	1,99	1,28	16,8	14,6	30,0	21,0	12,6	0,03	0,16	0,45	0,23	0,02	0,34
Sente-se fragilizado	5,46	1,36	1,09	24,2	28,8	28,0	9,8	3,7	-0,06	0,07	0,68	0,33	-0,01	0,36
Risco de demissão	5,24	1,41	1,14	21,6	34,6	23,1	9,0	6,5	0,00	0,07	0,50	0,24	-0,04	0,26
Tratamento autoritário	4,80	1,07	1,10	38,3	25,1	21,0	7,9	2,9	-0,06	0,01	0,63	0,32	-0,02	0,32
Sentir-se substituível	4,80	1,92	1,36	17,5	21,7	25,6	12,3	18,1	0,04	0,11	0,65	0,32	0,03	0,33
Remuneração														
Despesas básicas	1,97	1,88	1,18	13,3	23,8	32,7	17,7	10,5	0,09	0,12	0,36	0,68	0,09	0,35
Despesas inesperadas	2,40	2,72	1,09	3,5	9,0	27,7	28,8	28,6	0,05	0,04	0,36	0,67	0,08	0,27
Ganho líquido	1,53	2,47	0,97	3,3	11,0	34,0	37,0	13,2	0,51	0,18	0,19	0,36	0,37	0,23
Direitos														
Aposentadoria	2,18	0,57	0,79	60,4	19,2	18,2	NA	NA	0,56	0,26	0,03	0,25	0,59	0,25
Indenização	2,84	0,93	0,85	38,4	27,3	31,5	NA	NA	0,25	0,16	0,11	0,21	0,44	0,23
Licença-Maternidade	2,40	0,34	0,62	71,4	18,5	7,6	NA	NA	0,48	0,08	-0,05	0,06	0,44	0,20
Seguro	2,18	0,49	0,64	57,2	33,0	7,6	NA	NA	0,38	0,13	-0,12	0,03	0,53	0,17
Exercício de Direitos														
Folga semanal	3,06	1,14	1,20	41,8	17,0	24,9	8,6	4,6	0,18	0,10	0,15	0,16	0,27	0,49
Atestado médico	3,06	1,46	1,36	36,0	14,0	21,4	17,5	8,0	0,21	0,12	0,27	0,22	0,22	0,56
Consulta médica	2,40	1,51	1,24	29,8	17,4	26,6	19,0	4,8	0,16	0,18	0,41	0,29	0,15	0,66
Férias	2,84	1,33	1,39	39,8	17,7	18,5	10,5	10,7	0,47	0,16	0,19	0,17	0,43	0,40
Folga por motivos pessoais	3,49	2,24	1,27	13,3	12,0	26,9	27,3	17,0	0,03	0,13	0,43	0,35	0,09	0,62
Folga por motivos familiares	3,71	2,12	1,25	14,5	13,5	28,2	26,4	13,7	0,03	0,08	0,42	0,32	0,07	0,62

Nota: correlações destacadas em negrito evidenciando cada conjunto de itens do respectivo construto teórico; n por item pode variar em função dados perdidos (*missing*).

DP: desvio padrão; NA: não se aplica.

A Tabela 4 mostra n, média, desvio padrão, proporção de dados perdidos, intervalos, piso e teto (%) e a consistência interna (alfa de Cronbach).

**Tabela 4:** Estatística descritiva das dimensões da versão brasileira da *Escala de Precariedade no Emprego* (EPRES-Br): média, desvio padrão (DP), amplitude (efeitos piso e teto) e consistência interna (alfa de Cronbach). Trabalhadores notificados pelo Sistema Único de Saúde (SUS) de Salvador e fisioterapeutas da Bahia, Brasil, 2023.

Dimensão	Itens da EPRES	n	Média	DP	Dados perdidos (%)	Intervalo	Piso (%)	Teto (%)	Alfa de Cronbach
Trabalhadores notificados pelo SUS									
Tempo de Permanência	1 e 2	166	1,29	1,01	7,8	0-4	17,2	1,7	0,43
Desempoderamento	3 e 4	176	2,11	1,15	2,2	0-4	13,9	6,1	0,75
Vulnerabilidade	5 a 9	174	1,29	0,89	3,3	0-4	5,6	0,6	0,73
Remuneração	10 a 12	178	2,46	1,06	1,1	0-4	1,1	7,8	0,79
Direitos	13 a 16	176	1,31	0,91	2,2	0-4	20,0	1,1	0,63
Exercício dos Direitos	17 a 22	177	1,34	0,99	0,1	0-4	17,2	0,6	0,77
EPRES-Br	1 a 22	158	1,62	0,55	12,2	0,52-3,04	0,6	0,6	0,77
Fisioterapeutas									
Tempo de Permanência	1 e 2	421	1,07	0,92	8,08	0-4	19,0	1,3	0,60
Desempoderamento	3 e 4	429	1,96	1,17	6,33	0-4	16,4	3,1	0,73
Vulnerabilidade	5 a 9	429	1,55	0,89	6,33	0-4	3,7	0,9	0,80
Remuneração	10 a 12	447	2,36	0,88	2,40	0-4	0,7	2,8	0,74
Direitos	13 a 16	442	1,17	1,06	3,49	0-4	30,8	2,6	0,70
Exercício dos Direitos	17 a 22	432	1,63	0,92	5,68	0-4	5,7	0,7	0,81
EPRES-Br	1 a 22	399	1,62	0,58	12,88	0,22-3,19	0,2	0,2	0,84

Nota: valores de alfa ≥ 0,70 foram considerados aceitáveis; piso e teto correspondem à proporção de respondentes nos valores mínimo e máximo da escala.

Entre os trabalhadores de Salvador, as subescalas variaram de 1,29 (Tempo de Permanência) até 2,46 (Remuneração). O escore global da EPRES-Br foi 1,62. O efeito piso apresentou valores abaixo de 5,7% em Vulnerabilidade e Remuneração, e registrou maiores proporções (> 13%) nas demais dimensões. O efeito teto foi geralmente baixo (< 5%), com exceção de Desempoderamento (6,1%) e Remuneração (7,8%). O coeficiente alfa de Cronbach esteve geralmente acima de 0,74, com exceção de Direitos (0,6) e Tempo de Permanência (0,5). O alfa global foi de 0,77 (Tabela 4) ^36^.

Entre os fisioterapeutas, as subescalas variaram de 1,07 (Tempo de Permanência) até 2,36 (Remuneração). O escore global da EPRES-Br também foi de 1,62. O efeito piso apresentou valores abaixo de 5,7% em Vulnerabilidade, Exercício dos Direitos e Remuneração, e registrou maiores proporções (> 16,38%) em Tempo de Permanência, Desempoderamento e Direitos. O efeito teto foi baixo (< 5%). O alfa de Cronbach esteve em 0,70 ou maior para todas as dimensões, exceto para Tempo de Permanência (0,60). O alfa global foi de 0,84 (Tabela 4) ^36^.

### Análise fatorial

A composição dos fatores e suas cargas são exibidas na Tabela 5. Nela, encontram-se também a proporção de variância por fator, alfa, índices H, RMSEA, CFI e TLI.

**Tabela 5:** Estrutura fatorial, consistência interna e estimativa de replicabilidade da versão brasileira da *Escala de Precariedade no Emprego* (EPRES-Br). Trabalhadores notificados pelo Sistema Único de Saúde (SUS) de Salvador e fisioterapeutas da Bahia, Brasil, 2023.

Item	Vulnerabilidade	Remuneração	Direitos	Desempoderamento	Exercício dos Direitos	Folga
5A. Trabalhadores notificados pelo SUS						
Duração do contrato			0,650			
Tempo trabalhado		0,340				
Definição de horário				0,639		
Definição de remuneração				0,913		
Exposto a punições			-0,352			
Sente-se fragilizado	0,752					
Risco de demissão	0,681					
Tratamento autoritário	0,823					
Sentir-se substituível	0,576					
Despesas básicas		0,764				
Despesas inesperadas		0,642				
Ganho líquido		0,914				
Aposentadoria			0,558			
Indenização			0,482			
Licença-Maternidade			0,707			
Seguro desemprego			0,755			
Folga semanal					0,508	
Atestado médico					0,756	
Consulta médica					0,634	0,356
Férias					0,732	
Folga por motivos pessoais						0,848
Folga por motivos familiares						0,906
Proporção da variância	0,230	0,140	0,100	0,060	0,080	0,050
Alfa de Cronbach	0,830	0,770	0,730	0,540	0,730	0,950
H-latente	0,865	0,878	0,843	0,872	0,851	0,896
H-observado	0,762	0,800	0,747	0,720	0,731	0,790
RMSEA	0,000					
CFI	0,999					
TLI	1,018					
5B. Fisioterapeutas						
Duração do contrato			0,807			
Tempo trabalhado			0,674			
Quem define horário				0,829		
Quem define remuneração				0,783		
Exposto a punições	0,491					
Sente-se fragilizado	0,882					
Risco de demissão	0,602					
Trato autoritário	0,846					
Sentir-se substituível	0,739					
Despesas básicas		0,959				
Despesas inesperadas		0,952				
Ganho líquido			0,547			
Aposentadoria			0,802			
Indenização			0,465			
Licença-Maternidade			0,773		0,351	
Seguro desemprego			0,714			
Folga semanal					0,543	
Atestado médico					0,795	
Consulta médica					0,586	
Férias			0,504		0,424	
Folga motivos pessoais						0,974
Folga motivos familiares						0,960
Proporção da variância	0,040	0,080	0,060	0,070	0,180	0,270
Alfa de Cronbach	0,830	0,950	0,830	0,910	0,730	0,980
H-latente	0,900	0,975	0,923	0,815	0,876	0,988
H-observado	0,919	1,002	0,829	0,707	0,848	1,029
RMSEA	0,099					
CFI	0,953					
TLI	0,905					

Nota: alfa de Cronbach ≥ 0,70 indica consistência interna aceitável. RMSEA com df = 114 para ambas amostras, IC95%: 0,0000-0,00331 para trabalhadores de Salvador e 0,0635-0,1143 para fisioterapeutas. Índice H > 0,80 indica fator bem definido. N = 158 para trabalhadores de Salvador e N = 399 para fisioterapeutas na Bahia. Cargas fatoriais apresentadas a partir de |0,30|. Cargas em negrito indicam retenção do item no fator; cargas sem negrito indicam cargas cruzadas não atribuídas a um fator específico.

CFI: índice de ajuste comparativo; RMSEA: erro quadrático médio de aproximação; TLI: índice de Tucker-Lewis.

Entre os trabalhadores de Salvador (n = 158), os itens apresentaram cargas fatoriais acima de 0,30 em seis fatores. O item “consulta médica” mostrou cargas cruzadas nos fatores Exercício dos Direitos e Folga, e as medidas de Pratt revelaram 0,67 para Exercício dos Direitos e 0,29 para Folga, retendo o item ao fator Exercício dos Direitos. A maioria dos itens carregou mais na sua dimensão teórica, exceto “duração do contrato” (Q1), “tempo trabalhado” (Q2), “exposto a punições” (Q5), “folga por motivos pessoas” (Q21) e “folga por motivos familiares” (Q22). Os seis fatores, acumulados, explicaram 67% da variância proporcional. O alfa foi acima de 0,73 para todos os fatores, exceto para Desempoderamento (0,54) (Tabela 5).

Entre os fisioterapeutas, todos os itens apresentaram cargas fatoriais acima de 0,35. Ocorreram cargas cruzadas no item “licença maternidade”, nos fatores Direitos e Exercício dos Direitos, para os quais as medidas de Pratt observadas foram 0,782 e 0,213, respectivamente, associando o mesmo ao fator Direitos. Além desse, “férias” carregou tanto em Direitos quanto em Exercício dos Direitos, com medidas de Pratt informando 0,528 para Direitos e 0,428 para Exercício dos Direitos, retendo o mesmo ao fator Direitos. A maioria dos itens apresentou a maior carga dentro da sua dimensão teórica, exceto “duração do contrato” (Q1), “tempo trabalhado” (Q2), “ganho líquido” (Q12), “folga por motivos pessoas” (Q21) e “folga por motivos familiares” (Q22). Esses seis fatores, acumulados, explicaram 71% da variância proporcional. A confiabilidade foi aceitável (acima de 0,70) para todos os fatores (Tabela 5).

## Discussão

A EPRES-Br apresentou, em ambas as amostras, boa performance, com pequena proporção de dados perdidos (boa aceitabilidade), utilização de todas as opções de respostas, além de uma confiabilidade da consistência interna global, a partir das dimensões do trabalho precário, satisfatória (alfa 0,77 e 0,84, respectivamente) ^37^. Os itens apresentaram boas propriedades psicométricas no contexto brasileiro, com correlação item-dimensão moderada (entre 0,4 e 0,59) ou forte (de 0,6 a 0,79) em quase todos os itens, e cargas fatoriais de moderadas a altas. Foi mantida a retenção de seis fatores, seguindo o modelo teórico, dada a consistência do construto que embasou a escala multidimensional que o operacionaliza, a EPRES ^19^. As dimensões do construto são plausíveis, mas apontamos ajustes necessários, especialmente nos quesitos 21 e 22 da dimensão Exercício dos Direitos. Os graus de liberdade (gl) de moderados a elevados (gl = 114) conferem mais solidez à confiabilidade do excelente ajuste observado na amostra de trabalhadores de Salvador, reduzindo a probabilidade de inflação do RMSEA. TLI > 1 revela modelos com ajustes elevados e estimativas sólidas e baseadas em *bootstrap* para intervalos de confiança, podendo ser superior a um, sendo considerado ajuste excelente, virtualmente interpretado como TLI = 1.

### Desempenho dos itens da EPRES

Embora a EPRES II-2010 tenha questões um pouco mais específicas para a vigência de contrato formal, adotamos algumas adaptações para o contexto com alta informalidade que representa a realidade contemporânea no mundo do trabalho. Discutimos aqui, no entanto, suas implicações.

As duas amostras registraram escore global médio de EPRES-Br 1,62. Esse valor é próximo dos encontrados em outras amostras internacionais: na América Central, EPRES 1,56, 10 itens ^21^; Suécia, EPRES-Se 1,9, 23 itens ^20^; Chile, EPRES-Ch 1,32, 21 itens ^22^; e Espanha, EPRES 1,7 e 1,6, 26 e 22 itens, respectivamente ^19^ ^,^ ^25^. Em outras subamostras da Espanha, EPRES 0,9 para ambas ^19^ ^,^ ^25^ e na amostra da Alemanha, EPRES-Ge 0,99, 22 itens ^23^, com predominância de trabalho padrão. Dessa forma, os níveis de precariedade obtidos nas amostras brasileiras foram mais elevados que aqueles com predominância de trabalho padrão ^19^ ^,^ ^20^ ^,^ ^21^ ^,^ ^22^ ^,^ ^23^ ^,^ ^25^. Apenas Vives et al. ^19^ utilizou o EPRES 2005, todos os demais utilizaram o EPRES 2010.

No entanto, embora sejam amostras com grande potencial de representatividade, comparações seriam imprudentes devido a contextos econômicos e legais diferentes dos países, perfil sociodemográfico e amostras não probabilísticas da maioria dos estudos, conforme exigido nos processos de validação, como discutem Reichenheim & Bastos ^35^.

Com os níveis de aceitabilidade acima de 90%, utilizando EPRES-Br para ambas as amostras deste estudo, obtivemos parâmetros compatíveis com os demais estudos de validação da EPRES: enquanto a maioria registrou aceitabilidade acima de 90%, apenas Suécia e Alemanha atingiram patamar acima de 95%. A confiabilidade da consistência interna nas amostras deste estudo esteve em patamares também compatíveis com os demais: Suécia 0,83, Chile 0,83 e Espanha 0,86 (2010) e 0,82 (2015); demonstrando desempenho igual ou superior em confiabilidade quando comparado com Alemanha (0,77) e América Central (0,5 para informais e 0,6 para formais) ^19^ ^,^ ^20^ ^,^ ^21^ ^,^ ^22^ ^,^ ^23^ ^,^ ^25^.

A proporção de respostas obtida para a amostra com trabalhadores de Salvador, 44,8%, está dentro de parâmetros encontrados para outros estudos com entrevistas à distância incluídos em metanálise, na qual foi encontrada 44.1% como proporção média de respostas ^38^.

Na dimensão Vulnerabilidade, o item “exposto a punições” apresentou, na amostra de trabalhadores de Salvador, correlação item-dimensão abaixo da média (Tabela 2) e carga negativa no fator Direitos, não se agregando ao fator Vulnerabilidade, correspondente à dimensão original (Tabela 5). Esse desempenho já foi observado anteriormente ^25^ com recomendação de exclusão do item do cálculo de confiabilidade para Vulnerabilidade ou adoção da formulação da EPRES I (2005). Um estudo chileno ^25^ também apontou como problemática a redação condicional. A formulação negativa pode dificultar a interpretação, sendo possível compreendê-la como “não ser retaliado”, um direito, justificando sua associação à dimensão direitos.

A dimensão Tempo de Permanência apresentou a menor consistência interna, atribuída ao item “duração de contrato”, refletindo em parte a correlação de fraca a moderada dos seus itens (Tabelas 2 e 3). Atribuímos esse achado a uma limitação das opções de respostas para o respondente autônomo, pouco representado nas respostas possíveis. Essa limitação se evidencia na maior proporção de dados ignorados para esse item em ambas as amostras (Tabelas 2 e 3). Em outros estudos, esse item já foi inconsistente, muito embora a solução supostamente promissora adotada na versão sueca, que incluiu na escala de respostas os itens “não tenho contrato” e “não sei”, não assegurou melhor desempenho ^20^. É boa prática evitar a ambiguidade na interpretação de questões por meio do cuidado no fraseamento dos itens ^37^.

### Méritos e limitações do estudo

Testar a EPRES-Br para mensurar a precariedade do trabalho no contexto brasileiro implicou fazê-lo incluindo não apenas trabalhadores em situação de emprego formal, mas, especialmente, aqueles sem estatuto de emprego, ou seja, trabalhadores informais ou com contratos temporários ou autônomos involuntários, como no caso dos fisioterapeutas, submetidos à corrosão das condições de trabalho. Assim sendo, optando por testar a EPRES-Br no mercado de trabalho contemporâneo, dada a realidade que se impõe, embora os resultados apresentados sejam promissores no sentido de recomendar a escala para mensurar o trabalho precário em sua multidimensionalidade, esses achados exigem parcimônia na sua interpretação.

Itens das dimensões Tempo de Permanência e Direitos carregando no mesmo fator, e itens sobre folgas (Q21 e Q22) carregando em fator próprio, afastado de Exercício dos Direitos, mostraram limitações. Diante disso, propõe-se a revisão desses itens, considerando o significado desses para o contexto do mundo do trabalho. Com as evidências dos diversos estudos conduzidos até aqui com a EPRES, é desejável avançar para a versão que reúna elementos das diferentes modalidades contratuais e da diversidade de cenários e relações de trabalho, contemporaneamente.

O presente estudo é o primeiro a publicar a EPRES II-2010 ^18^ traduzida e adaptada para o Português, no Brasil, sendo o primeiro a avaliar as propriedades psicométricas da EPRES-Br. Além disso, o estudo utilizou duas amostras heterogêneas de trabalhadores, compostas, ambas, de trabalhadores permanentes e temporários, assegurando boa variabilidade de exposição ao trabalho precário.

O uso de uma EFA flexível, com base no software FACTOR, que a considera como inscrita nos modelos de equações estruturais (SEM), permitiu que procedimentos sofisticados como RDWLS, RMSEA, CFI e TLI pudessem ser utilizados para avaliar a estrutura fatorial da EPRES-Br ^34^.

Elencamos, porém, algumas limitações do estudo. O escore global da EPRES-Br foi de 1,62, nesta escala com escore máximo de 4,0, o que nos faz indagar sobre a sua sensibilidade para identificar com maior precisão as condições efetivas de exposição ao trabalho precário.

Buscamos comparar a população estudada de fisioterapeutas com outro estudo com amostra de conveniência com esta categoria, que mostrou perfil sociodemográfico semelhante: média de 32 anos de idade; pós-graduados, 79,4%; e mulheres, 67% ^39^. Portanto, não há elementos que apontem diferenças significativas entre os respondentes e os não respondentes. E também, virtualmente, todos poderiam ter recebido o convite.

Obtivemos em ambas as amostras valores de H-latente ligeiramente superiores aos H-observados, indicando que os fatores são mais estáveis em nível teórico (latente) quando comparado à sua operacionalização empírica. Mesmo assim, a convergência entre ambos é adequada e essa diferença é comum com dados psicométricos, notadamente com itens ordinais ^29^.

Assinala-se como uma escolha adotada neste estudo a inclusão de benefícios sociais (como o Bolsa Família) nos “ganhos líquidos”, admitindo-se que este auxílio pode ter efeito sobre o trabalho precário, haja vista que tal benefício pode atenuar a subordinação a situações mais precarizadas de trabalho, o que talvez tenha repercutido nas respostas ao item ^40^. Essa adaptação encontra paralelo no estudo sueco que incluiu gorjetas ao ganho líquido, admitindo o papel de outras fontes de ganhos, com vistas à maior fidedignidade da renda auferida, na mesma perspectiva que adotamos ^19^ ^,^ ^21^. Ademais, parece-nos pertinente essa adaptação, ainda que possa limitar a comparabilidade direta com outros estudos que utilizaram apenas “ganhos líquidos”.

A EPRES-Br apresentou desempenho razoável no contexto brasileiro, tendo uma confiabilidade e uma aceitabilidade satisfatórias. Alguns resultados divergentes do arranjo das dimensões do construto original, neste estudo de adaptação transcultural, são apresentados, bem como algumas hipóteses explicativas.

As propriedades psicométricas das dimensões vulnerabilidade, desempoderamento e remuneração reiteram a escala original e contribuem, como estão formuladas e traduzidas, para a mensuração de trabalho precário no Brasil, e as demais (tempo de permanência, exercício de direitos e direitos) podem ser objeto de ajuste na formulação dos itens, a fim de oferecerem mensurações discriminativas à luz do construto original.

A EPRES visa a operacionalizar um construto teórico sobre relações de poder no emprego e no local de trabalho, buscando a mensuração de condições existentes de trabalho e riscos percebidos por trabalhadores. No contexto contemporâneo, é importante destacar, estão relegados às piores condições os jovens, as mulheres, os imigrantes e os trabalhadores racializados ^1^.

Nunes et al. ^41^ traduziram e fizeram um estudo de validação da EPRES proposta por Creed et al. ^42^, destinada a mensurar a precariedade laboral como “precariedade subjetiva”, originalmente aplicada à avaliação do trabalho precário desempenhado por estudantes universitários. A EPRES, por sua vez, visa a relações de poder no emprego e no local de trabalho, extraindo informações sobre aspectos contratuais, condições e relações de trabalho ^6^. Ademais, seu retrospecto de validação em 11 países inspira confiança para a aplicabilidade e a adaptabilidade em diferentes contextos.

A aplicação da EPRES-Br em trabalhadores com diferentes modalidades de vínculo laboral, incluindo inserções informais no mercado de trabalho, o que reflete mais de perto a contemporaneidade, constitui um uso para além das relações de emprego assalariado. Essa ampliação do escopo pode influenciar a forma como determinadas dimensões colaboram na mensuração da precariedade e deve ser interpretada com a cautela que uma adaptação exige.

É preciso que o construto trabalho precário, em sua multidimensionalidade, seja crescentemente mensurado em estudos epidemiológicos, de modo a produzir mais evidências acerca da determinação social de saúde e doença entre trabalhadores. A ampliação da erosão das condições no mundo do trabalho contemporâneo requer a abordagem do trabalho precário e das dimensões que o compõem, a fim de melhor explicá-lo, tendo em perspectiva apoiar políticas públicas, visando à regulação de práticas de gestão do trabalho pelas empresas e à identificação das relações de trabalho ambíguas ou disfarçadas ^3^.

## Data Availability

Os dados de pesquisa não estão disponíveis.

## Material Suplementar

Material Suplementar
