# Supplementary material for: ERRATUM: Measurement of precarious work in Brazil: translation, adaptation and dimensionality of the Employment Precariousness Scale (EPRES-Br)
Source: Cad Saude Publica. 2026 Nov 9;42:eER206125. doi: 10.1590/0102-311XEREN206125 (PMC13750637; doi:10.1590/0102-311XEREN206125)
Supplement: Translation

# Translation: ERRATUM: Mensuração do trabalho precário no Brasil: tradução, adaptação e dimensionalidade da *Escala de Precariedade do Emprego* (EPRES-Br).

**DOI:** 10.1590/0102-311XERPT206125

**Santana VR, Vives-Vergara A, Jesus GRS, Fernandes RCP. Mensuração do trabalho precário no Brasil: tradução, adaptação e dimensionalidade da *Escala de Precariedade do Emprego* (EPRES-Br). Cad Saúde Pública 2026; 42:e00206125.**

Onde se lê:

Victor Rocha Santana ^1^

Alejandra Vives-Vergara ^2^

Gláucio Roberto Santana de Jesus ^3^

Rita de Cássia Pereira Fernandes ^3^

^1^ Instituto de Saúde Coletiva, Universidade Federal da Bahia, Salvador, Brasil.

^2^ Universidad Católica de Chile, Santiago, Chile.

^3^ Programa de Pós-graduação em Saúde, Ambiente e Trabalho, Universidade Federal da Bahia, Salvador, Brasil.

Correspondência

V. R. Santana

Instituto de Saúde Coletiva, Universidade Federal da Bahia.

Rua Basílio da Gama s/n, Campus Universitário Canela, Salvador, BA 40110-040, Brasil.

vilarongavictor@gmail.com

Leia-se:

Victor Rocha Santana ^1^

Alejandra Vives-Vergara ^2^

Gláucio Roberto Santana de Jesus ^3^

Rita de Cássia Pereira Fernandes ^1,3^

^1^ Programa de Pós-graduação em Saúde Coletiva, Universidade Federal da Bahia, Salvador, Brasil.

^2^ Escuela de Salud Pública, Pontificia Universidad Católica de Chile, Santiago, Chile

^3^ Programa de Pós-graduação em Saúde, Ambiente e Trabalho, Universidade Federal da Bahia, Salvador, Brasil.

*Correspondência

V. R. Santana

Programa de Pós-graduação em Saúde Coletiva, Universidade Federal da Bahia.

Rua Basílio da Gama s/n, Campus Universitário Canela, Salvador, BA 40110-040, Brasil.

vilarongavictor@gmail.com

Onde se lê:

Colaboradores

V. R. Santana contribuiu com a concepção do estudo, análise e interpretação dos dados e redação; e aprovou a versão final. A. Vives-Vergara contribuiu com a concepção do estudo, redação e revisão; e aprovou a versão final. G. R. S. Jesus contribuiu com a concepção do estudo, redação e revisão; e aprovou a versão final. R. C. P. Fernandes contribuiu com a concepção do estudo, redação e revisão; e aprovou a versão final.

Leia-se:

Colaboradores

V. R. Santana contribuiu com a concepção do estudo, coleta, análise, interpretação dos dados e redação; e aprovou a versão final. A. Vives-Vergara contribuiu com a interpretação dos dados e revisão; e aprovou a versão final. G. R. S. Jesus contribuiu com a coleta de dados e revisão; e aprovou a versão final. R. C. P. Fernandes contribuiu com a concepção do estudo, análise e interpretação dos dados, redação e revisão; e aprovou a versão final.

Responsáveis pela aprovação:

Editora-chefe Marilia Sá Carvalho (0000-0002-9566-0284)

Editora-chefe Luciana Dias de Lima (0000-0002-0640-8387)

Editora-chefe Luciana Correia Alves (0000-0002-8598-4875)
